# Dynamic coupling effects of geometric eccentricity on multi-stage gear transmission via stiffness modulation and error excitation

**DOI:** 10.1038/s41598-025-33768-z

**Published:** 2025-12-29

**Authors:** Wenjia Lu, Guangda Liang, Zunling Du, Weibo Huang, Xiaoyu Zhao

**Affiliations:** College of Mechanical and Automotive Engineering, Zhaoqing University, Zhaoqing, 526061 China

**Keywords:** Multi-stage gear transmission, Geometric eccentricity, Stiffness modulation, Error excitation, Dynamic behavior, Engineering, Physics

## Abstract

This research investigates the dynamic mechanism arising from the coupling between multi-path stiffness modulation and geometric eccentricity in multi-stage gear transmission systems (MGTS) for high-end equipment. The study aims to address the critical challenges in vibration suppression and precision control for such systems. A lumped parameter model integrating translational and rotational degrees of freedom was established, and an analytical method for three-path stiffness modulation based on center distance fluctuation, pressure angle reconstruction, and contact ratio transition was proposed. The influence of geometric eccentricity on the dynamic characteristics via the combined action of stiffness excitation and displacement error was then systematically analyzed. It was found that a strong nonlinear coupling exists between the eccentricity ratio and the initial phase difference: the combination of extreme asymmetry and an anti-phase condition markedly amplifies the stiffness modulation amplitude, inducing severe vibration, while symmetric eccentricity coupled with phase optimization effectively mitigates these oscillations. The feasibility of the established model was verified experimentally. The simulated and measured frequency spectra showed good agreement at the meshing frequency and its major sidebands, thereby demonstrating the accuracy of the stiffness-error coupling mechanism. Ultimately, a dynamic performance-based “parameter design safety domain” was constructed, which explicitly defines a low-vibration design interval centered on the eccentricity ratio and phase difference. This work thereby not only deepens the theoretical understanding of the error-stiffness-vibration coupling mechanism in the MGTS but also provides theoretical support and a practical guide for the dynamic design, vibration suppression, and operational optimization of high-precision gear transmission systems.

## Introduction

The gear transmission system serves as the central power transfer unit in various high-end equipment, including wind turbines, aircraft engines, industrial robots, and high-precision numerical control machine tool, and its dynamic performance is critical to the machine’s reliability, operational efficiency, and service lifetime^1–3^. Gear transmission systems universally exhibit multiple internal excitations due to practical factors such as manufacturing tolerances, assembly conditions, and load-induced deformations. Geometric eccentricity, a typical error originating from manufacturing and assembly processes, has become a key factor influencing the system’s dynamic performance^4,5^. Under high-speed and heavy-load conditions, geometric eccentricity alters the mesh stiffness and center distance, thereby inducing strong parametric excitation that causes the system’s vibrational energy to concentrate around the mesh frequency and its sidebands. This excitation not only amplifies noise and dynamic load amplitudes but also leads to periodic fluctuations in tooth contact stress, accelerating the initiation of failure modes such as fatigue pitting and tooth breakage. These effects underscore the significant impact of geometric eccentricity in practical engineering and highlight the importance of further in-depth research.

In studies of gear systems, scholars conducted systematic research on the modeling and influence mechanisms of geometric eccentricity in different types of gear pairs, laying an important foundation for understanding gear system dynamics. Research on fixed-axis gear trains has primarily focused on dynamic modeling of geometric eccentricity and the analysis of parametric excitation mechanisms^6–11^. By establishing dynamic models for fixed-axis gear pairs that integrate time-varying mesh stiffness, transmission error, and dynamic backlash, studies in^6–8^ have revealed a mechanism whereby geometric eccentricity, by changing the position of the line of action and contact conditions, excites intense parametric excitation. The work in^9,10^ provided a new approach for condition monitoring of high-precision gear systems. Starting from a frequency-domain energy viewpoint, it constructed a quantitative mapping of geometric eccentricity to vibrational response, proposed a diagnostic method based on extracting fault-sensitive features from frequency-domain characteristics, and achieved early detection of micron-scale eccentricity. Reference^11^ developed a comprehensive dataset with multiple fault severity levels, achieving continuous adjustment of fault severity through a novel eccentricity simulation structure. By employing spectral correlation analysis and deep learning techniques, the study demonstrated the reliability of the data in characterizing fault features and its response under variable conditions, thereby providing high-quality training and validation resources for data-driven models in gear eccentricity fault diagnosis.

In the area of error modeling for planetary gear systems (PGS), research has primarily focused on the influence of manufacturing errors on system vibrational characteristics, modulation sidebands, and load-sharing behavior. Through the development of a coupled dynamic model that accounts for floating components and time-varying mesh stiffness in the PGS, the studies in^12,13^ systematically elucidated how eccentric errors propagate and couple within the complex mesh interactions, thereby establishing a theoretical basis for transmission error analysis in multi-planet systems. Regarding the interaction between errors and load-sharing behavior in the PGS, scholars have employed quasi-static contact mechanics models^14^, nonlinear dynamic models^15^, and three-dimensional planetary load distribution models^16^ to systematically analyze the effects of various error sources. These include carrier pinhole position errors, gear eccentricity, and run-out. Research demonstrates that these errors not only introduce displacement excitation by altering mesh positions but also generate inertial excitation, ultimately leading to uneven load sharing among planets and increased transmission error^17^. At the experimental level, the significant influence of errors on gear stress and load distribution has been further verified through methods such as strain measurement^18^. These studies consistently demonstrate that manufacturing errors are a critical factor affecting the load performance of the PGS. In the field of fault diagnosis, studies^19,20^ have focused on the intelligent identification of early-stage geometric eccentricity and local faults in planetary gears. The research revealed the characteristic excitation patterns of different fault types (such as cracks and spalls) in transmission error and vibration responses, and achieved precise fault localization by leveraging meshing phase relationships. This methodology has been experimentally validated, providing an effective solution for early fault diagnosis and intelligent maintenance of the PGS under strong noise conditions.

These findings deepen the understanding of PGS dynamics and the multi-path excitation mechanism of errors like eccentricity, providing a foundation for subsequent research on the dynamic design, vibration suppression, and condition monitoring of multi-stage transmission systems.

With the widespread application of multi-stage compound transmission structures in high-end equipment-such as the hybrid configuration of “parallel fixed-axis stage and planetary stage” commonly used in wind turbine gearboxes and the multi-stage planetary gear compound configurations in tunnel boring machines-the internal excitation sources within systems have become more diverse, and the dynamic coupling behaviors have become particularly complex. Building on research into single-stage systems, scholars have increasingly recognized that the cross-scale dynamic coupling effects induced by manufacturing errors, such as geometric eccentricity, in multi-stage transmissions have become a critical factor affecting transmission performance^21–27^. Existing research indicates that certain progress has been made in the dynamic modeling and error excitation analysis of MGTS. Studies^21,22^ have established a load-sharing coefficient calculation model considering multiple errors and a dynamic model of a two-stage PGS, respectively, revealing the significant influence of manufacturing and assembly errors on the system’s load-sharing performance. Studies^23,24^ further indicate that carrier assembly errors and gear geometric eccentricity can significantly alter the dynamic mesh force and vibration response of the system, and may even induce changes in the frequency structure. In the areas of fault coupling and system regulation, studies^25–27^ have all been dedicated to developing dynamic models for MGTS that integrate fault-induced excitation and stiffness modulation, aiming to reveal the cross-scale influence mechanisms of errors and defects in compound transmissions.

Collectively, these studies demonstrate that fault and error behaviors in MGTS are highly coupled, necessitating holistic modeling and coordinated regulation from multiple dimensions such as stiffness excitation, load transmission, and system elasticity. They also provide an important reference for investigating the coupled dynamic mechanisms of geometric eccentricity in MGTS. Nevertheless, a unified theoretical framework and a deep mechanistic understanding are still lacking regarding the error coupling mechanism between parallel and planetary stages, the cooperative evolution of stiffness, and their impact on the overall dynamic behavior of the system, which points to an important direction for future research.

In current research, several underlying coupling mechanisms still require further revelation. The respective contributions of the multiple pathways through which geometric eccentricity jointly modulates mesh stiffness — namely center distance fluctuation, pressure angle reconstruction, and contact ratio variation — have not yet been systematically analyzed. Furthermore, the coupling relationship between the base circle displacement error and tooth profile error induced by eccentricity still needs precise modeling. In MGTS, a quantitative description of the mechanism by which the phase difference between eccentricities in different gears suppresses or amplifies vibration sidebands is lacking, making it difficult to support tolerance design and dynamic optimization for high-precision gear systems.

In response to the aforementioned challenges, and building upon existing research, this paper is dedicated to establishing a closed-loop coupling dynamics theory of “stiffness-error” interactions in MGTS. The work will focus intensively on the following aspects: developing a multi-path analytical model for stiffness modulation under the influence of geometric eccentricity, systematically revealing the coupled influence mechanisms of center distance fluctuation, pressure angle reconstruction, and contact ratio variation on time-varying stiffness, and quantifying the contribution weight of each physical path; elucidating the nonlinear joint regulatory mechanism of eccentricity amplitude ratio and phase difference on the vibration response in MGTS, with particular attention to the suppression effect of specific phase combinations on vibration sideband energy. This research is expected to provide a theoretical basis and technical support for precision design, dynamic optimization, and condition monitoring of high-end equipment transmission systems.

## Dynamic modeling of MGTS under geometric eccentricity excitation

### System structure and definition of degrees of freedom

**Fig. 1:**
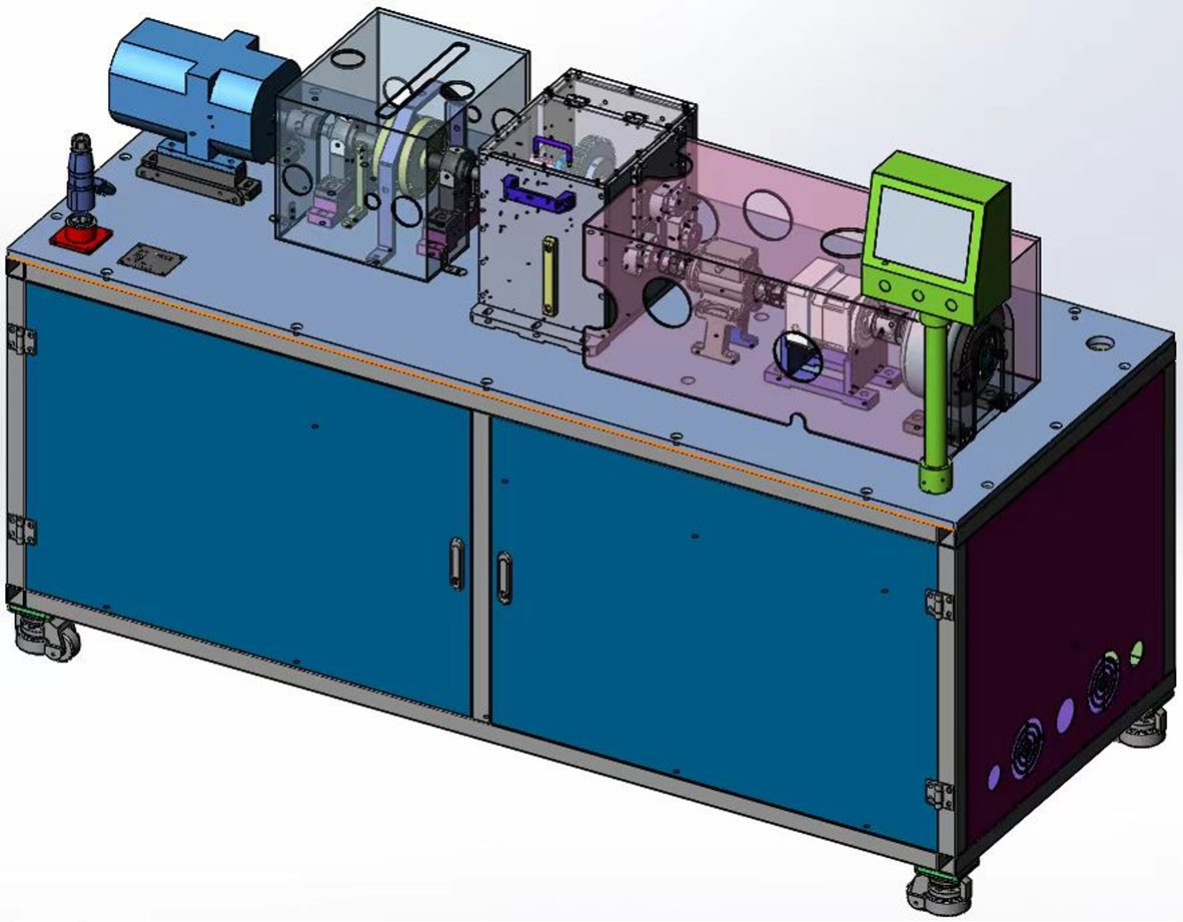
3D model of the multi-stage gear transmission system test rig.

**Fig. 2:**
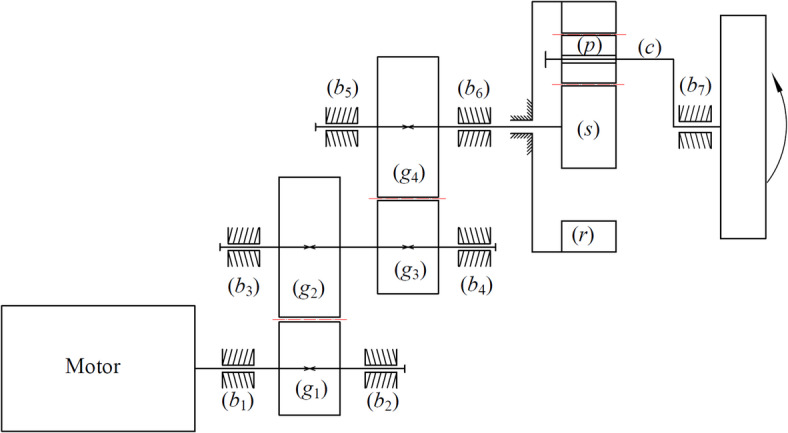
Kinematic schematic of the multi-stage gear transmission system.

The study focuses on a compound MGTS comprising a two-stage fixed-axis gear transmission and a single-stage PGS. Its three-dimensional experimental bench model and kinematic diagram are shown in Figs. 1 and 2, respectively.

The power flow path of the system is illustrated in Fig. 2: The motor is connected to the pinion (*g*_1_) of the first-stage fixed-axis transmission, and the input shaft carrying *g*_1_ is defined as Shaft 1. *g*_1_ meshes with the first large gear (*g*_2_), and this stage is defined as the first-stage gear transmission. *g*_2_ is rigidly connected to the pinion (*g*_3_) of the second-stage fixed-axis transmission via Shaft 2, establishing a series power flow. *g*_3_ meshes with the large gear (*g*_4_), and this stage is defined as the second-stage gear transmission. *g*_4_ is connected to the sun (*s*) of the PGS via the output shaft (Shaft 3), transmitting power to the PGS, which is defined as the third-stage gear transmission. In the PGS, the sun gear (*s*) acts as the power input, while the carrier (*c*) serves as the power output and is connected to the external load. The ring gear (*r*) is fixed to the housing. The system employs three planet gears (*p*_*n*_, *n* = 1, 2, 3) evenly distributed between the sun and ring to achieve power splitting. The bearings at various stages are denoted as *b*_*i*_ (*i* = 1, 2, …, 7).

### Multi-scale coupled dynamic modeling of the system

To accurately characterize the dynamic behavior of the system under geometric eccentricity excitation, this study employs a lumped parameter method to establish a coupled translational-torsional dynamic model. In this model, each component is assigned three degrees of freedom: two translational degrees of freedom (*x*, *y*) along mutually perpendicular directions, and one equivalent torsional displacement degree of freedom (*u*). Here, the equivalent torsional displacement is defined as *u* = *r*_*b*_⋅*θ*, where *r*_*b*_ represents the base circle radius of the gear and *θ* denotes the absolute angular torsional displacement. This definition of degrees of freedom effectively couples the lateral and torsional vibrations of the components, thereby establishing a foundation for subsequent analysis of the system’s complex dynamic response under excitation from multiple error sources.

**Fig. 3:**
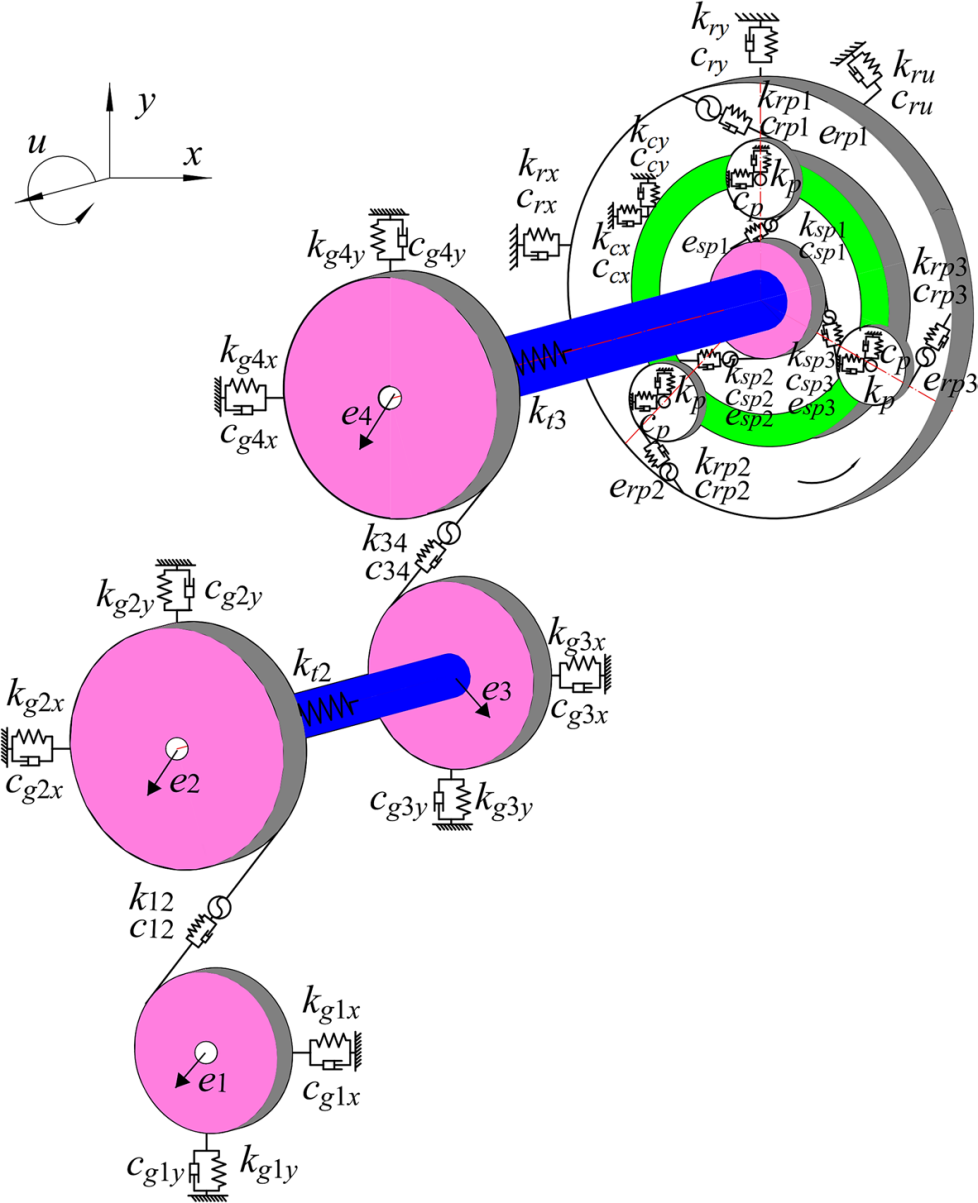
Translational-torsional coupled dynamic model of the MGTS.

The translational-torsional coupled dynamic model of the MGTS established in this study is shown in Fig. 3. This model integrates modeling approaches from Zhang et al.^28^ for fixed-axis gear pairs and from Lin and Parker^29^ for the PGS.

The model fully incorporates elastic coupling between components, damping effects, and key excitation from geometric eccentricity. The specific parameter definitions are as detailed below:

(1) Stiffness parameters:

Bearing support stiffness: *k*_*gix*_, *k*_*giy*_ (*i* = 1, 2, 3, 4) denote the support stiffness of the fixed-axis gears *g*_*i*_ in the *x* and *y* directions, respectively; *k*_*jx*_, *k*_*jy*_ (*j* = *c*, *r*, *s*, *p*) represent the support stiffness of the various components within the PGS.

Mesh stiffness of gear pairs: The mesh stiffness of gear pair *g*_1_-*g*_2_ is denoted by *k*_12_, and that of gear pair *g*_3_-*g*_4_ is denoted by *k*_34_. For the PGS, the mesh stiffness between the sun and each planet is denoted by *k*_*spn*_ (*n* = 1, 2, 3), and the mesh stiffness between the ring and each planet is denoted by *k*_*rpn*_ (*n* = 1, 2, 3).

Inter-stage coupling stiffness: The coupling stiffness between the first and second stages is defined as *k*_*t*2_, and the coupling stiffness between the second and third stages is defined as *k*_*t*3_.

(2) Damping parameters: The naming convention for all damping parameters in the model corresponds exactly to that of the stiffness parameters. For example, *c*_*gix*_, *c*_*giy*_ (*i* = 1, 2, 3, 4) denote the support damping of the fixed-axis gears *g*_*i*_ in the *x* and *y* directions, respectively; *c*_*jx*_, *c*_*jy*_ (*j* = *c*, *r*, *s*, *p*) represent the support damping of the various components within the PGS. The mesh damping of gear pair *g*_1_-*g*_2_ is denoted by *c*_12_, and that of gear pair *g*_3_-*g*_4_ is denoted by *c*_34_. In the PGS, the mesh damping between the sun and each planet, between the ring and each planet, are denoted by *c*_*spn*_ and *c*_*rpn*_ (*n* = 1, 2, 3), respectively.

(3) Error excitation parameters: The geometric eccentricity error of *g*_*i*_ is denoted as *e*_*i*_ (*i* = 1, 2, 3, 4). The static transmission errors of gear pairs *g*_1_-*g*_2_ and *g*_3_-*g*_4_ induced by geometric eccentricity are denoted as *e*_ecc,12_ and *e*_ecc,34_, respectively. The static transmission errors between the sun and each planet, and between the ring and each planet, induced by geometric eccentricity, are denoted as *e*_*spn*_ and *e*_*rpn*_ (*n* = 1, 2, 3), respectively.

The equations of motion for the MGTS can be expressed as:

1 $${\mathbf{M\ddot {q}}}\left( t \right)+{\mathbf{C\dot {q}}}\left( t \right)+{\mathbf{Kq}}\left( t \right)={{\mathbf{F}}_{{\mathrm{ext}}}}+{{\mathbf{F}}_{{\mathrm{ecc}}}}(t)$$

where: **M** denotes the system mass matrix, **q**(*t*) represents the generalized coordinate vector of the system (comprising 10 core components with 30 degrees of freedom), **K** is the system stiffness matrix, **C** is the system damping matrix, formulated as Rayleigh damping **C** = *α***M** + *β***K**^30^, **F**_ext_ signifies the external load vector, and **F**_ecc_(*t*) indicates the eccentric excitation vector.

The mass matrix of the MGTS is given by:

2 $${\mathbf{M}}={\mathrm{diag}}\left[ {{{\mathbf{M}}_c},{{\mathbf{M}}_r},{{\mathbf{M}}_s},{{\mathbf{M}}_{p1}},{{\mathbf{M}}_{p2}},{{\mathbf{M}}_{p3}},{{\mathbf{M}}_{g1}},{{\mathbf{M}}_{g2}},{{\mathbf{M}}_{g3}},{{\mathbf{M}}_{g4}}} \right]$$

The stiffness matrix consists of five sub-matrices:

3 $${\mathbf{K}}={{\mathbf{K}}_{{\mathrm{bearing}}}}+{{\mathbf{K}}_{{\mathrm{shaft}}}}+{{\mathbf{K}}_{{\mathrm{mesh}}}}+{{\mathbf{K}}_{{\mathrm{planet}}}}+{{\mathbf{K}}_{{\mathrm{couple}}}}$$

The bearing support stiffness matrix **K**_bearing_ can be expressed as:

4 $${{\mathbf{K}}_{{\mathrm{bearing}}}}={\mathrm{diag[}}{{\mathbf{K}}_c}{\mathrm{,}}{{\mathbf{K}}_r}{\mathrm{,}}{{\mathbf{K}}_s}{\mathrm{,}}{{\mathbf{K}}_{p1}}{\mathrm{,}}{{\mathbf{K}}_{p2}}{\mathrm{,}}{{\mathbf{K}}_{p3}}{\mathrm{,}}{{\mathbf{K}}_{g1}}{\mathrm{,}}{{\mathbf{K}}_{g2}}{\mathrm{,}}{{\mathbf{K}}_{g3}}{\mathrm{,}}{{\mathbf{K}}_{g4}}{\mathrm{]}}$$

where: the support stiffness sub-matrices within the PGS are given by **K**_*j*_ = diag [*k*_*jx*_, *k*_*jy*_, 0] (*j* = *c*, *r*, *s*, *p*_*n*_); the support stiffness sub-matrices in the fixed-axis transmission are given by **K**_*gi*_ = diag [*k*_*gix*_, *k*_*giy*_, 0] (*i* = 1, 2, 3, 4).

The torsional stiffness **K**_shaft_ of the shaft is written as:

5 $${{\mathbf{K}}_{{\mathrm{shaft}}}}={{\mathbf{K}}_{t2}}+{{\mathbf{K}}_{t3}}$$

The torsional stiffness matrix **K**_*t*2_ for Shaft 2 can be written as:

6 $${{\mathbf{K}}_{t2}}=\left[ {\begin{array}{*{20}{c}} {\mathbf{0}}&{\mathbf{0}}& \vdots & \vdots &{\mathbf{0}} \\ {}&{{{\mathbf{K}}_{{\mathrm{24,24}}}}}& \cdots &{{{\mathbf{K}}_{24,27}}}&{} \\ \vdots & \vdots & \ddots & \vdots & \vdots \\ {}&{{{\mathbf{K}}_{27,24}}}& \cdots &{{{\mathbf{K}}_{27,27}}}&{\mathbf{0}} \\ {\mathbf{0}}& \vdots & \vdots &{\mathbf{0}}& \ddots \end{array}} \right]\begin{array}{*{20}{c}} ,&{\left\{ {\begin{array}{*{20}{l}} {{{\mathbf{K}}_{24,24}}={\mathrm{diag}}[0,0,\frac{{{k_{t2}}}}{{r_{{b2}}^{2}}}]} \\ {{{\mathbf{K}}_{27,27}}={\mathrm{diag}}[0,0,\frac{{{k_{t2}}}}{{r_{{b3}}^{2}}}]} \\ {{{\mathbf{K}}_{24,27}}={{\mathbf{K}}_{27,24}}={\mathrm{diag}}[0,0, - \frac{{{k_{t2}}}}{{{r_{b2}}{r_{b3}}}}]} \end{array}} \right.} \end{array}$$

The torsional stiffness matrix **K**_*t*3_ for Shaft 3 can be written as:

7 $${{\mathbf{K}}_{t3}}=\left[ {\begin{array}{*{20}{c}} {\mathbf{0}}&{\mathbf{0}}& \vdots & \vdots &{\mathbf{0}} \\ {}& \ddots & \cdots &{}& \vdots \\ \vdots & \vdots &{{{\mathbf{K}}_{{\mathrm{9,9}}}}}& \vdots &{{{\mathbf{K}}_{9,30}}} \\ {\mathbf{0}}&{}& \cdots & \ddots & \vdots \\ {\mathbf{0}}&{\mathbf{0}}&{{{\mathbf{K}}_{30,9}}}& \cdots &{{{\mathbf{K}}_{30,30}}} \end{array}} \right],\left\{ {\begin{array}{*{20}{l}} {{{\mathbf{K}}_{9,9}}={\mathrm{diag}}[0,0,\frac{{{k_{t3}}}}{{r_{{bs}}^{2}}}]} \\ {{{\mathbf{K}}_{30,30}}={\mathrm{diag}}[0,0,\frac{{{k_{t3}}}}{{r_{{b4}}^{2}}}]} \\ {{{\mathbf{K}}_{9,30}}={{\mathbf{K}}_{30,9}}={\mathrm{diag}}[0,0, - \frac{{{k_{t3}}}}{{{r_{bs}}{r_{b4}}}}]} \end{array}} \right.$$

The mesh stiffness matrix **K**_mesh_ for the fixed-axis gear pairs can be expressed as

8 $${{\mathbf{K}}_{{\mathrm{mesh}}}}={{\mathbf{K}}_{12}}+{{\mathbf{K}}_{34}}$$

In Eq. (8), the mesh stiffness matrix **K**_12_ for gear pair *g*_1_-*g*_2_ is:

9 $$\begin{gathered} {{\mathbf{K}}_{12}}={k_{12}}{{\mathbf{D}}_{12}}{\mathbf{D}}_{{12}}^{{\mathrm{T}}}\begin{array}{*{20}{c}} ,&{{{\mathbf{D}}_{12}}={{\left[ {{{\mathbf{0}}_{1 \times 18}}, - \cos {\alpha _{12}}, - \sin {\alpha _{12}},1,\cos {\alpha _{12}},\sin {\alpha _{12}},1,{{\mathbf{0}}_{1 \times 6}}} \right]}^{\mathrm{T}}}} \end{array} \hfill \\ \hfill \\ \end{gathered}$$

In Eq. (8), the mesh stiffness matrix **K**_34_ for gear pair *g*_3_-*g*_4_ is:

10 $${{\mathbf{K}}_{34}}={k_{34}}{{\mathbf{D}}_{34}}{\mathbf{D}}_{{34}}^{T}\begin{array}{*{20}{c}} ,&{{{\mathbf{D}}_{34}}=[{{\mathbf{0}}_{1 \times 24}}} \end{array}, - \cos {\alpha _{34}}, - \sin {\alpha _{34}},1,\cos {\alpha _{34}},\sin {\alpha _{34}},1{]^{\mathrm{T}}}$$

The mesh stiffness matrix **K**_planet_ of the PGS can be expressed as:

11 $${{\mathbf{K}}_{{\mathrm{planet}}}}=\sum\limits_{{n=1}}^{3} {\left( {{\mathbf{K}}_{{sp}}^{n}+{\mathbf{K}}_{{rp}}^{n}} \right)}$$

In Eq. (11), the sun-planet mesh stiffness matrix is:

12 $${\mathbf{K}}_{{sp}}^{n}={k_{spn}}{\mathbf{D}}_{{sp}}^{n}{({\mathbf{D}}_{{sp}}^{n})^{\mathrm{T}}}{\begin{array}{*{20}{c}} ,&{{\mathbf{D}}_{{sp}}^{n}=\left[ {{{\mathbf{0}}_{1 \times 6}}, - \cos {\psi _n}, - \sin {\psi _n}, - 1,{{\mathbf{0}}_{1 \times (3+9(n - 1))}},\cos {\psi _n},\sin {\psi _n},1,{{\mathbf{0}}_{1 \times (9 - 3n)}}} \right]} \end{array}^{\mathrm{T}}}$$

where: *ψ*_*n*_ = *ϕ*_*n*_ + *α*_*spn*_, and *ϕ*_*n*_ = (*n* − 1)(2*π*/3). Here, *ϕ*_*n*_ is the planetary gear phase angle, and *α*_*spn*_ is the pressure angle of the sun-planet mesh.

In Eq. (11), the ring-planet mesh stiffness matrix is:

13 $${\mathbf{K}}_{{rp}}^{n}={k_{rpn}}{\mathbf{D}}_{{rp}}^{n}{({\mathbf{D}}_{{rp}}^{n})^{\mathrm{T}}}\begin{array}{*{20}{c}} ,&{{\mathbf{D}}_{{rp}}^{n}={{\left[ {{{\mathbf{0}}_{1 \times 3}},\cos {\gamma _n},\sin {\gamma _n}, - 1,{{\mathbf{0}}_{1 \times (6+9(n - 1))}}, - \cos {\gamma _n}, - \sin {\gamma _n},1,{{\mathbf{0}}_{1 \times (12 - 3n)}}} \right]}^{\mathrm{T}}}} \end{array}$$

where: *γ*_*n*_ = *ϕ*_*n*_ − *α*_*rpn*_, and *α*_*rpn*_ is the pressure angle of the ring-planet mesh.

The carrier-planet coupling stiffness matrix **K**_couple_ can be expressed as:

14 $${{\mathbf{K}}_{{\mathrm{couple}}}}=\sum\limits_{{n=1}}^{3} {\left( {{k_{cx}}{Q_n}Q_{n}^{{\mathrm{T}}}+{k_{cy}}{{\mathbf{S}}_n}{\mathbf{S}}_{n}^{{\mathrm{T}}}} \right)}$$

The coefficient matrices ***Q***_*n*_ and ***S***_*n*_ in Eq. (14) can be expressed as:

15 $$\left\{ {\begin{array}{*{20}{c}} {{Q_n}={{\left[ {1,0, - {r_c}\sin {\phi _n},{{\mathbf{0}}_{1 \times 6}}, - {\delta _{n1}}, - {\delta _{n2}}, - {\delta _{n3}},{{\mathbf{0}}_{1 \times (21 - 3n)}}} \right]}^{\mathrm{T}}}} \\ {{{\mathbf{S}}_n}={{\left[ {0,1,{r_c}\cos {\phi _n},{{\mathbf{0}}_{1 \times 6}},0, - {\delta _{n1}}, - {\delta _{n2}}, - {\delta _{n3}},{{\mathbf{0}}_{1 \times (21 - 3n)}}} \right]}^{\mathrm{T}}}} \end{array} } \right.$$

where: *δ*_*ij*_ denotes the Kronecker function.

The time-varying mesh deformation of gear pair *g*_1_-*g*_2_ resulting from geometric eccentricity is given by:

16 $$\left\{ \begin{gathered} {e_{{\mathrm{ecc}},12}}\left( t \right)={e_1}\cos \left( {\frac{{{u_{g1}}\left( t \right)}}{{{r_{b1}}}} - {\alpha _{12}}} \right) - {e_2}\cos \left( {\frac{{{u_{g2}}\left( t \right)}}{{{r_{b2}}}} - {\alpha _{12}}} \right) \hfill \\ {{\dot {e}}_{{\mathrm{ecc}},12}}\left( t \right)= - \frac{{{e_1}{{\dot {u}}_{g1}}}}{{{r_{b1}}}}\sin \left( {\frac{{{u_{g1}}\left( t \right)}}{{{r_{b1}}}} - {\alpha _{12}}} \right)+\frac{{{e_2}{{\dot {u}}_{g2}}}}{{{r_{b2}}}}\sin \left( {\frac{{{u_{g2}}\left( t \right)}}{{{r_{b2}}}} - {\alpha _{12}}} \right) \hfill \\ \end{gathered} \right.$$

The resulting eccentric excitation is:

17 $${{\mathbf{F}}_{{\mathrm{ecc}},12}}\left( t \right)=\left[ {{k_{12}}\left( t \right){e_{{\mathrm{ecc}},12}}\left( t \right)+{c_{12}}\left( t \right){{\dot {e}}_{{\mathrm{ecc}},12}}\left( t \right)} \right]{{\mathbf{D}}_{12}}$$

The time-varying mesh deformation of gear pair *g*_3_-*g*_4_ resulting from geometric eccentricity is given by:

18 $$\left\{ \begin{gathered} {e_{{\mathrm{ecc}},34}}\left( t \right)={e_3}\cos \left( {\frac{{{u_{g3}}\left( t \right)}}{{{r_{b3}}}} - {\alpha _{34}}} \right) - {e_4}\cos \left( {\frac{{{u_{g4}}\left( t \right)}}{{{r_{b4}}}} - {\alpha _{34}}} \right) \hfill \\ {{\dot {e}}_{{\mathrm{ecc}},34}}\left( t \right)= - \frac{{{e_3}{{\dot {u}}_{g3}}}}{{{r_{b3}}}}\sin \left( {\frac{{{u_{g3}}\left( t \right)}}{{{r_{b3}}}} - {\alpha _{34}}} \right)+\frac{{{e_4}{{\dot {u}}_{g4}}}}{{{r_{b4}}}}\sin \left( {\frac{{{u_{g4}}\left( t \right)}}{{{r_{b4}}}} - {\alpha _{34}}} \right) \hfill \\ \end{gathered} \right.$$

The resulting eccentric excitation is:

19 $${{\mathbf{F}}_{{\mathrm{ecc}},34}}\left( t \right)=\left[ {{k_{34}}\left( t \right){e_{{\mathrm{ecc}},34}}\left( t \right)+{c_{34}}\left( t \right){{\dot {e}}_{{\mathrm{ecc}},34}}\left( t \right)} \right]{{\mathbf{D}}_{34}}$$

The total eccentric excitation generated by the fixed-axis gear system, **F**_ecc_(*t*), is:

20 $${{\mathbf{F}}_{{\mathrm{ecc}}}}\left( t \right)={{\mathbf{F}}_{{\mathrm{ecc}},12}}\left( t \right)+{{\mathbf{F}}_{{\mathrm{ecc}},34}}\left( t \right)$$

### Modulation mechanism of time-varying mesh stiffness

**Fig. 4:**
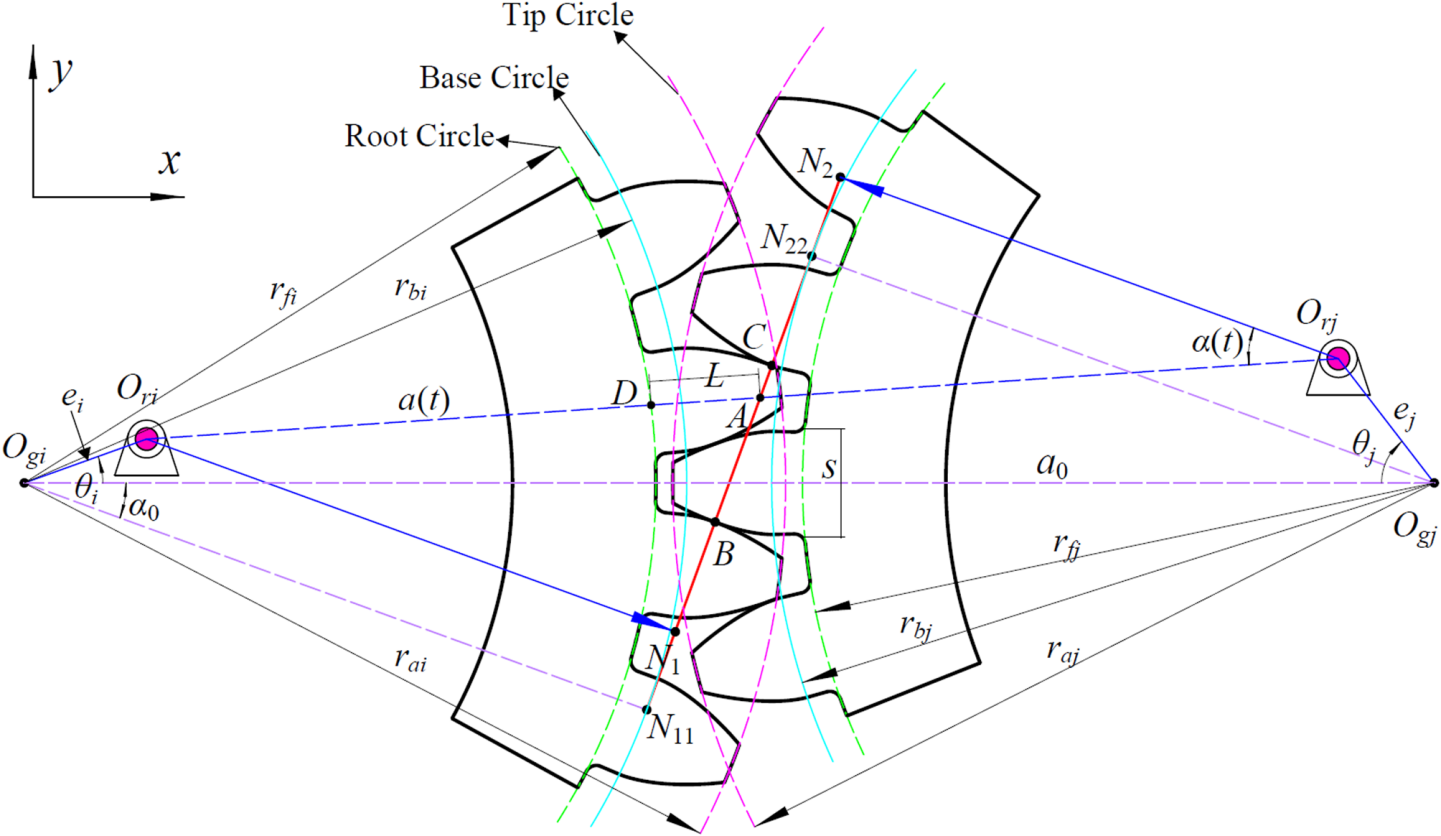
Eccentricity-mesh model of gear pair *g*_*i*_-*g*_*j*_.

The stiffness modulation model established in this section is based on the small eccentricity assumption (*e*/*a*_0_ < 0.01), which aligns with the precision requirements of most engineering applications and ensures the validity of the subsequent linearization approximation. To characterize the modulation effects and coupling mechanisms of dual rotational frequencies (Ω*i*, Ω*j*) in a gear pair (*g*_*i*_-*g*_*j*_) with geometric eccentricities (eccentricities *e*_*i*_, *e*_*j*_), a model for time-varying mesh stiffness modulation induced by geometric eccentricity was developed. The eccentricity-mesh model for gear pair *g*_*i*_-*g*_*j*_ is illustrated in Fig. 4, where: the geometric centers of gears *g*_*i*_ and *g*_*j*_ are located at *O*_*gi*_ and *O*_*gj*_, respectively; their actual rotation centers are at *O*_*ri*_ and *O*_*ri*_, respectively; the coordinate system is defined with *O*_*gi*_ as the origin and the direction *O*_*gi*_*O*_*gj*_ as the *x*-axis.

The geometric eccentricities of gears *g*_*i*_ and *g*_*j*_ are denoted as *e*_*i*_ and *e*_*j*_, respectively. The eccentricity phase angles are *θ*_*i*_ and *θ*_*j*_, respectively. The nominal center distance of the gear pair is *a*_0_, and the nominal pressure angle is *α*_0_. Due to the presence of geometric eccentricity, these parameters become time-varying: the time-varying center distance induced by geometric eccentricity is defined as *a*(*t*), and the time-varying pressure angle is defined as *α*(*t*). Furthermore, geometric eccentricity alters the start and end points of the mesh, thereby affecting the contact ratio of the gear pair. The theoretical line of action for gear pair *g*_*i*_-*g*_*j*_ is *N*_11_*N*_22_, while the actual line of action becomes *N*_1_*N*_2_. The root circle radii of gear pair *g*_*i*_-*g*_*j*_ are *r*_*fi*_ and *r*_*fj*_, respectively; the base circle radii are *r*_*bi*_ and *r*_*bj*_, respectively; and the tip circle radii are *r*_*a.i.*_ and *r*_*aj*_, respectively.

#### Time-varying model of center distance and pressure angle

The rotation center position of gear *g*_*i*_:

21 $$\left\{ {\begin{array}{*{20}{c}} {{x_i}={e_i}\cos ({\Omega _i}t+{\phi _i})} \\ {{y_i}={e_i}\sin ({\Omega _i}t+{\phi _i})} \end{array}} \right.$$

The rotation center position of gear *g*_*j*_:

22 $$\left\{ {\begin{array}{*{20}{l}} {{x_j}={a_0}+{e_j}\cos ({\Omega _j}t+{\phi _j})} \\ {{y_j}={e_j}\sin (\Omega {}_{j}t+{\phi _j})} \end{array}} \right.$$

where: Ω_*i*_ and Ω_*j*_ represent the rotational angular velocities of the gear pair, and *φ*_*i*_ and *φ*_*j*_ denote the initial phase angles.

The actual center distance *a*(*t*) of the gear pair can be expressed as:

23 $$a(t)=\sqrt {{{({x_j} - {x_i})}^2}+{{({y_j} - {y_i})}^2}}$$

Expanding and retaining first-order small quantities (*e*_*i*_ ≪ *a*_0_, *e*_*j*_ ≪ *a*_0_):

24 $$a(t) \approx {a_0}+\Delta a(t)={a_0}+{e_j}\cos \left( {{\Omega _j}t+{\phi _j}} \right) - {e_i}\cos \left( {{\Omega _i}t+{\phi _i}} \right)$$

Therefore, the fluctuation Δ*a*(*t*) of the center distance can be written as:

25 $$\Delta a(t)={e_j}\cos \left( {{\Omega _j}t+{\phi _j}} \right) - {e_i}\cos \left( {{\Omega _j}t+{\phi _j}} \right)={e_j}\cos {\theta _j} - {e_i}\cos {\theta _i},\begin{array}{*{20}{c}} {}&{{\theta _l}=\frac{{{u_{gl}}\left( t \right)}}{{{r_{bl}}}}} \end{array} \left( {l=i,j} \right)$$

Since the base circle radii are constant, the actual pressure angle *α*(*t*) satisfies:

26 $$\cos \alpha (t)=\frac{{{r_{bi}}+{r_{bj}}}}{{a(t)}}$$

Taylor expansion to the first-order term yields:

27 $$\alpha (t) \approx {\alpha _0}+\Delta \alpha (t)\begin{array}{*{20}{c}} ,&{} \end{array}\Delta \alpha (t)=\frac{{\cot {\alpha _0}}}{{{a_0}}}\Delta a(t)$$

Substituting into Eq. (25) yields:

28 $$\Delta \alpha (t)= \frac{{\cot {\alpha _0}}}{{{a_0}}} ({e_j}\cos {\theta _j} - {e_i}\cos {\theta _i})$$

#### 2.3.2 Three-path modulation mechanism of mesh stiffness

The mesh stiffness *k*_*ij*_(*t*) of *g*_*i*_-*g*_*j*_ can be defined as^13^:

29 $${k_{ij}}(t)={k_{avg}}+\Delta {k_e}(t)$$

where: *k*_*avg*_ denotes the average mesh stiffness, and Δ*k*_*e*_(*t*) represents the stiffness modulation term induced by eccentricity errors.

Eccentricity leads to uneven load distribution among tooth pairs, resulting in variations in contact deformation and consequently, changes in contact stiffness. The altered pressure angle due to eccentricity causes the mesh point to shift along the tooth profile height, thereby modifying the bending stiffness of the gear tooth. Simultaneously, the change in center distance induces a variation in the contact ratio, which may lead to a jump in the number of teeth in contact.

Based on the above mechanistic analysis, the stiffness modulation term Δ*k*_*e*_(*t*) induced by eccentricity can be decomposed into three components:

30 $$\Delta {k_e}=\Delta {k_{{\mathrm{load}}}}+\Delta {k_b}+\Delta {k_\varepsilon }$$

where: Δ*k*_load_ is the stiffness variation induced by the load redistribution effect, Δ*k*_*b*_ is the stiffness variation caused by the mesh point displacement, and Δ*k*_*ε*_ is the stiffness variation resulting from contact ratio fluctuation.

a) Effect of load redistribution.

The total mesh force *F*_total_ required to transmit the same torque *T*_*i*_ changes as follows:

31 $${F_{{\mathrm{total}}}}(t)=\frac{{{T_i}}}{{{r_{bi}}+{e_i}cos{\theta _i}cos{\alpha _0}}}$$

Since e_*i*_ ≪ *r*_*bi*_, *F*_total_ can be approximated expanded as:

32 $${F_{\mathrm{t}\mathrm{o}\mathrm{t}\mathrm{a}\mathrm{l}}}(t) \approx {F_0}\left( {1 - {\lambda _i}{e_i}\cos {\theta _i}} \right), {\lambda _i} = \frac{{\cos {\alpha _0}}}{{{r_{bi}}}}$$

where: *F*_0_ denotes the rated load under design conditions.

The load share on the *m*-th tooth pair is:

33 $${F_m}(t)=[\frac{1}{{{N_c}(t)}} +{\kappa _m}(t)] {F_{{\mathrm{total}}}}(t)$$

where: *N*_c_(*t*) denotes the instantaneous number of tooth pairs in contact, and *κ*_*m*_(*t*) is the load distribution deviation caused by eccentricity:

34 $${\kappa _m}(t)= \frac{1}{{{g_{\alpha 0}}}} [{e_i}\cos {\theta _i} - {e_j}\cos {\theta _j})], {g_{\alpha 0}}={a_0}\tan {\alpha _0}$$

where *g*_*α*0_ represents the theoretical length of the line of action. *κ*_*m*_(*t*) is the load distribution deviation on the *m*-th tooth pair caused by eccentricity, which is proportional to the difference in the eccentricity projections along the line of action.

According to Hertz contact theory, when the tooth surfaces are approximated as smooth spherical surfaces, *k*_*h, m*_ ∝ *F*_*m*_^1/3^^31^. Consequently, the contact stiffness under an arbitrary load *F*_*m*_ is given by:

35 $${k_{h,m}}={k_{h0}} {(\frac{{{F_m}}}{{{F_0}}})^{1/3}}$$

where *k*_*h*0_ represents the nominal value of the contact stiffness.

The load varies due to geometric eccentricity. Defining the actual load as *F*_*m*_=*F*_0_ + Δ*F*_*m*_, where Δ*F*_*m*_ is the deviation from the rated load, the resulting variation in contact stiffness is thus given by:

36 $$\Delta {k_{h,m}}={k_{h,m}} - {k_{h0}}={k_{h0}} \left[ {{{(1+\frac{{\Delta {F_m}}}{{{F_0}}})}^{1/3}} - 1} \right]$$

When the condition Δ*F*_*m*_ / *F*_0_ ≪ 1 holds true, a Taylor series expansion is applied, and by neglecting the higher-order terms, we obtain:

37 $$\Delta {k_{h,m}} \approx {k_{h0}}\left( {1+\frac{1}{3} \frac{{\Delta {F_m}}}{{{F_0}}} - 1} \right)=\frac{{{k_{h0}}}}{3} \frac{{\Delta {F_m}}}{{{F_0}}}$$

Therefore, the variation in contact stiffness induced by load redistribution is:

38 $$\Delta {k_{{\mathrm{load}}}}(t)=\frac{1}{{{N_c}}}\sum\limits_{{m=1}}^{{{N_c}}} {\Delta {k_{h,m}}} =\frac{{{k_{h0}}}}{{3{F_0}}} \cdot \frac{1}{{{N_c}}}\sum\limits_{{m=1}}^{{{N_c}}} {\Delta {F_m}}$$

b) Effect of mesh point position shift.

Bending deformation is induced by the tangential component of the force, *F*_*t*_ = *F*_total_ cos *α*. The bending deformation of the gear tooth can be expressed as:

39 $${\delta _b}=\frac{{{F_t}{L^3}}}{{3EI}}=\frac{{{F_{{\mathrm{total}}}}\cos \alpha \cdot {L^3}}}{{3E \cdot \frac{{b{s^3}}}{{12}}}}=\frac{{4{F_{{\mathrm{total}}}}\cos \alpha \cdot {L^3}}}{{Eb{s^3}}}$$

where: *E* represents the elastic modulus of the gear material, *I* denotes the moment of inertia of the critical cross-section, *b* is the face width of the gear, *L* signifies the distance from the meshing point to the tooth root, and *s* is the tooth thickness at the root critical section.

Thus, the bending stiffness of the gear tooth can be approximately expressed as:

40 $${k_b}\left( L \right)=\frac{{F\cos \alpha }}{{{\delta _b}}}=\frac{{Eb{s^3}}}{{4{L^3}}}$$

The sensitivity of the bending stiffness to the force arm is:

41 $$\frac{{{\mathrm{d}}{k_b}}}{{{\mathrm{d}}L}}= - \frac{3}{L}{k_b}$$

The first-order approximation of the variation in bending stiffness can be expressed as:

42 $$\Delta {k_b} \approx \frac{{{\mathrm{d}}{k_b}}}{{{\mathrm{d}}L}}\Delta L= - \frac{{3{k_b}}}{L}\Delta L$$

Differentiating the radial distance *L* from the meshing point to the root critical section, given by *L* = *r*_*b*_ / cos *α* – *r*_*f*_, yields:

43 $$\frac{{{\mathrm{d}}L}}{{{\mathrm{d}}\alpha }}={r_b} \cdot \frac{{\sin \alpha }}{{{{\cos }^2}\alpha }}$$

Therefore, the variation in the force arm *L* caused by a change in the pressure angle Δ*α* is given by:

44 $$\Delta L \approx {\left. {\frac{{{\mathrm{d}}L}}{{{\mathrm{d}}\alpha }}} \right|_{{\alpha _0}}}\Delta \alpha ={r_b}\frac{{\sin {\alpha _0}}}{{{{\cos }^2}{\alpha _0}}}\Delta \alpha$$

Substituting the expression for Δ*L* into Eq. (42) yields:

45 $$\Delta {k_b} \approx - \frac{{3{k_{b0}}}}{{{L_0}}}\left( {{r_b}\frac{{\sin {\alpha _0}}}{{{{\cos }^2}{\alpha _0}}}\Delta \alpha } \right)$$

Subsequently, substituting the expression for the pressure angle variation Δ*α* into the equation for Δ*k*_*b*_ yields:

46 $$\Delta {k_b}(t)= - \frac{{3{k_{b0}}{r_b}}}{{{a_0}{L_0}\cos {\alpha _0}}}\left( {{e_j}\cos {\theta _j} - {e_i}\cos {\theta _i}} \right)$$

where: *k*_*b*_ denotes the nominal value of the gear bending stiffness.

c) Effect of contact ratio fluctuation.

The expression for the length of the line of action can be formulated as:

47 $${g_\alpha }(t)=\sqrt {r_{{ai}}^{2} - r_{{bi}}^{2}} +\sqrt {r_{{aj}}^{2} - r_{{bj}}^{2}} - a(t)\sin \alpha (t)$$

Neglecting higher-order infinitesimals, Eq. (47) can be further expressed as:

48 $${g_\alpha }(t) \approx {g_{\alpha 0}} - \sin {\alpha _0} \cdot \Delta a(t) - {a_0}\cos {\alpha _0} \cdot \Delta \alpha (t)$$

Substituting Δ*α*(*t*) yields:

49 $${g_\alpha }(t) \approx {g_{\alpha 0}} - \frac{{\Delta a(t)}}{{\sin {\alpha _0}}}$$

Therefore, the expression for the contact ratio can be written as:

50 $$\varepsilon (t)=\frac{{{g_\alpha }(t)}}{{{p_b}}} \approx {\varepsilon _0} - \frac{{\Delta a(t)}}{{{p_b}\sin {\alpha _0}}}$$

where *p*_*b*_ is the base pitch. Substituting Eq. (25) into the expression for the contact ratio yields:

51 $$\varepsilon (t)={\varepsilon _0}+{\eta _e}({e_i}\cos {\theta _i} - {e_j}\cos {\theta _j}), {\eta _e}=\frac{1}{{{p_b}\sin {\alpha _0}}}$$

The meshing tooth number transition function of the gear pair is expressed as:

52 $${N_c}\left( t \right)=\left\{ {\begin{array}{*{20}{l}} {\left\lfloor {{\varepsilon _0}} \right\rfloor {\mathrm{if}}\cos \left[ {\pi \left( {\varepsilon \left( t \right) - \left\lfloor {{\varepsilon _0}} \right\rfloor } \right)} \right] \geqslant 0 } \\ {\left\lfloor {{\varepsilon _0}} \right\rfloor +1 {\mathrm{otherwise}}} \end{array}} \right.$$

where the term cos [π(*ε* − *n*)] = cos (π*δ*) serves as a phase function determining the meshing state: when *δ* < 0.5, cos (π*δ*) > 0, the number of tooth pairs in contact remains *n*; when *δ* ≥ 0.5, cos(π*δ*) ≤ 0, the number of tooth pairs transitions to *n* + 1.

Therefore, the meshing stiffness transition function of the gear pair can be expressed as:

53 $$\Delta {k_\varepsilon }=\frac{{{k_0}}}{{{N_{c0}}}}\left( {{N_c}\left( t \right) - {N_{c0}}} \right)$$

For the stiffness modulation term Δ*k*_*e*_(*t*) = *f*(*e*_*i*_ cos *θ*_*i*_, *e*_*j*_ cos *θ*_*j*_), when the eccentricity is small (*e*/*a*_0_ < 0.01), its first-order Taylor series expansion is given by:

54 $$\Delta {k_e}(t)={\left. {\frac{{\partial \Delta {k_e}}}{{\partial {e_i}}}} \right|_{{e_i}=0}}e{}_{i}\cos {\theta _i}+{\left. {\frac{{\partial \Delta {k_e}}}{{\partial {e_j}}}} \right|_{{e_j}=0}}{e_j}\cos {\theta _j}+O({e^2})$$

The eccentricity-stiffness coupling coefficient *β*_*l*_ is defined as:

55 $${\beta _l}={\left. {\frac{{\partial \Delta {k_e}}}{{\partial {e_l}}}} \right|_{{e_l}=0}} \left( {l=i,j} \right)$$

The stiffness modulation term Δ*k*_*e*_(*t*) can be further expressed as:

56 $$\Delta {k_e}(t) \approx {\beta _i}{e_i}\cos {\theta _i}+{\beta _j}{e_j}\cos {\theta _j}$$

The eccentricity-stiffness coupling coefficient can be further expressed as:

57 $${\beta _l}=\underbrace {{\frac{{{k_{h0}}}}{{3{r_{bl}}}} \cos {\alpha _0}}}_{{\beta _{l}^{{{\mathrm{load}}}}}}+\underbrace {{{{\left( { - 1} \right)}^{l+1}} \cdot \frac{{3{k_{b0}}{r_{bl}}}}{{{a_0}{L_0}\cos {\alpha _0}}}}}_{{\beta _{l}^{b}}}+\underbrace {{\frac{{{k_0}}}{{N_{{c0}}^{2}{p_{bl}} \sin {\alpha _0}}} \cdot {\delta _{\varepsilon n}}}}_{{\beta _{l}^{\varepsilon }}} , \left( {l=i,j} \right)$$

where: *δ*_*εn*_ is defined as the activation factor for the contact ratio transition term, and *η*_*c*_ is the critical deviation threshold that determines whether the contact ratio transition is activated, which is dependent on the gear accuracy level, $${\delta _{\varepsilon n}}=\left\{ {\begin{array}{*{20}{l}} {1 |{\varepsilon _0} - n|<{\eta _c}} \\ {0 {\mathrm{otherwise}}} \end{array}} \right.$$.

## The influence mechanism of eccentricity on excitation characteristics

The core parameters of the MGTS dynamic model established in this study are defined collectively by the PGS parameters in Table 1, the fixed-axis gear train parameters in Table 2, and the meshing and coupling stiffness parameters in Table 3. Tables 1 and 2 provide comprehensive design parameters, such as the number of teeth, module, and pressure angle, along with the gear mass and equivalent moment of inertia, thereby establishing a data foundation for the coupling of inertial effects and eccentricity-induced excitations.

Equivalent moment of inertia (*I*/*r*_*b*_^2^): The gear’s moment of inertia *I* is normalized (unit: kg) such that the torsional degree of freedom *u* = *r*_*b*_*θ* becomes dimensionally consistent with the translational degrees of freedom. This enables consistent modeling of the coupled translational-torsional dynamic equations (as detailed in Sect. Multi-scale coupled dynamic modeling of the system).

Equivalent circumferential torsional stiffness (*k*_*eq*_ = *k*_*t*_/*r*_*b*_^2^): The torsional stiffness is converted into a physical quantity dimensionally consistent with the translational stiffness (unit: N/m), ensuring that the torsional and translational terms in the stiffness matrix can be directly superimposed.

**Table 1:** Parameters of the PGS.

Gear designation	Sun	Planet	Ring	Carrier
Number of teeth	9	36	81	-
Module (mm)	1.5	1.5	1.5	-
Pressure angle (°)	20	20	20	-
Mass (kg)	0.107	0.224	1.273	0.975
Equivalent moment of inertia (kg)	0.0938	0.112	0.265	0.214
Radial support stiffness (N/m)	1.5 × 10^8^	1.5 × 10^8^	1.5 × 10^8^	1.5 × 10^8^
Equivalent torsional stiffness (N/m)	2.0 × 10^8^	2.0 × 10^8^	2.0 × 10^8^	2.0 × 10^8^

**Table 2:** Parameters of two-stage fixed-axis gear train.

Gear designation	g_1_	g_2_	g_3_	g_4_
Number of teeth	25	58	28	60
Module (mm)	2	2	2	2
Pressure angle (°)	20	20	20	20
Mass (kg)	0.221	1.087	0.271	1.164
Equivalent moment of inertia (kg)	0.112	0.272	0.135	0.291
Radial support stiffness (N/m)	1.5 × 10^8^	1.5 × 10^8^	1.5 × 10^8^	1.5 × 10^8^
Equivalent torsional stiffness (N/m)	2.0 × 10^8^	2.0 × 10^8^	2.0 × 10^8^	2.0 × 10^8^

**Table 3:** Mean values of meshing stiffness and coupling stiffness between components.

Stiffness designation	Meshing stiffness (sun-planet)	Meshing stiffness (ring-planet)	Meshing Stiffness (g_1_-g_2_)	Meshing Stiffness (g_3_-g_4_)	Coupling Stiffness (g_2_-g_3_)	Coupling Stiffness (g_4_-s)
Value (N/m)	2.4 × 10^8^	3.8 × 10^8^	4.2 × 10^8^	4.4 × 10^8^	2.0 × 10^8^	2.0 × 10^8^

### Single-parameter sensitivity and phase modulation

**Fig. 5:**
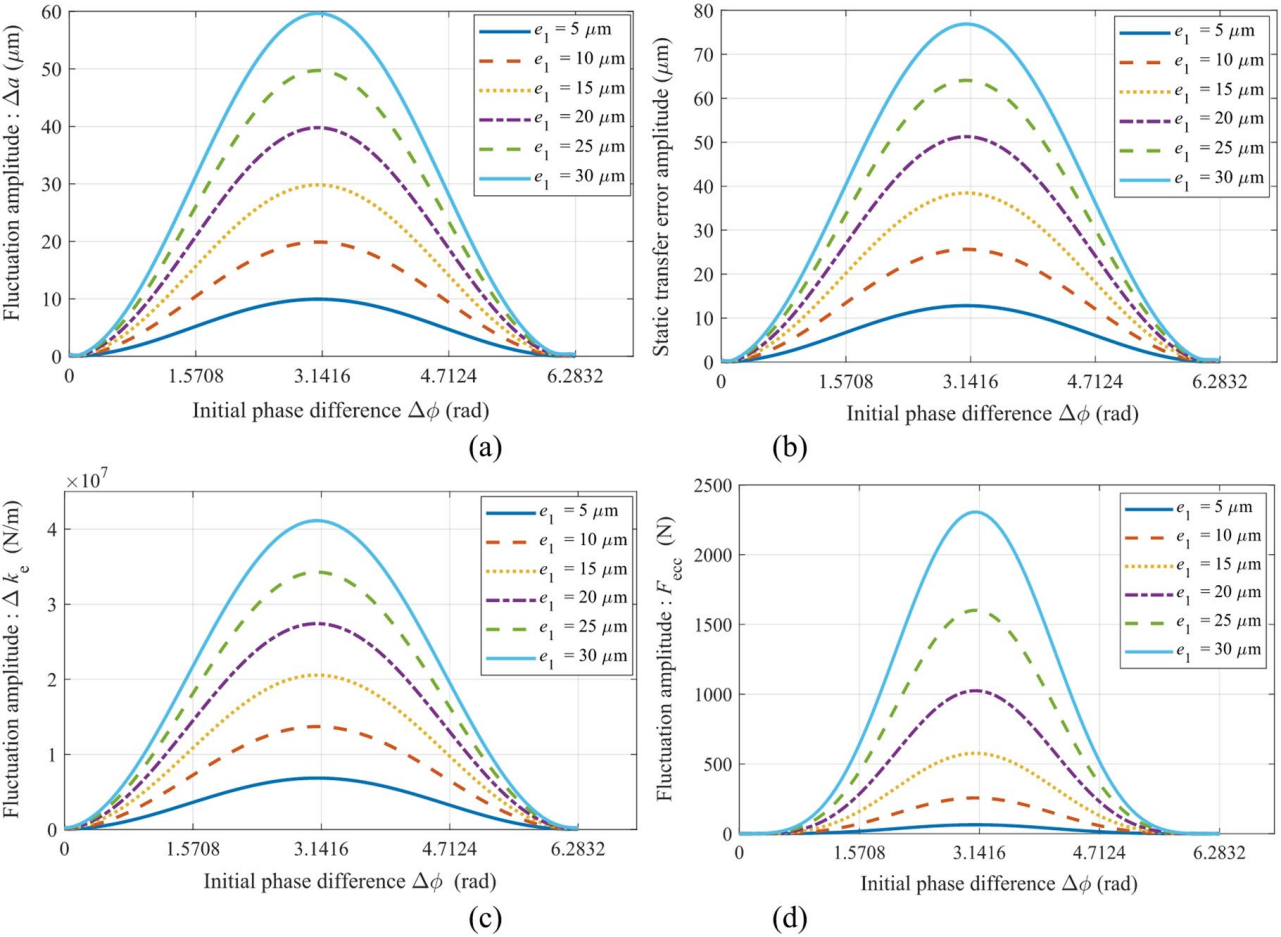
Mapping relationship between the initial phase difference and dynamic excitation parameters of gear pair *g*_1_-*g*_2_ with variation of the single parameter *e*_1_: (**a**) amplitude of center distance fluctuation, (**b**) static transmission error, (**c**) mesh stiffness modulation term, (**d**) amplitude of eccentric excitation fluctuation.

Figure 5 illustrates the relationship between the initial phase difference of gear pair *g*_1_-*g*_2_ and dynamic excitation parameters when varying the amplitude of parameter *e*_1_ (with an eccentricity ratio *e*_1_/*e*_2_ set to 1, and without considering eccentricity errors in *e*_3_ and *e*_4_): (a) amplitude of center distance fluctuation, (b) static transmission error, (c) meshing stiffness modulation term, and (d) amplitude of coupled eccentricity excitation fluctuation.

Figure 5 illustrates how the initial phase difference modulates the dynamic excitation of gear pair *g*_1_-*g*_2_:

(1) The center distance fluctuation amplitude Δ*a* (Fig. 5a) peaks at Δ*ϕ* = π and minimizes at Δ*ϕ* = 0 or 2π. Under the condition of Δ*φ* = π, Δ*a* shows a linear positive correlation with the amplitude of e1: Δ*a* = 10 μm when *e*_1_ = 5 μm, and increases to 60 μm when *e*_1_ = 30 μm.

(2) The amplitude of the static transmission error varies approximately sinusoidally with Δ*ϕ*, reaching its maximum at Δ*ϕ* = π (78 μm when *e*_1_ = 30 μm) and its minimum near Δ*ϕ* = 0 and 2π.

(3) The stiffness modulation term Δ*k*_*e*_ increases significantly at Δ*ϕ* = π (Δ*k*_*e*_ ≈ 4.2 × 10⁷ N/m when *e*_1_ = 30 μm) and reaches its minimum (close to zero) at Δ*ϕ* = 0 and 2π, demonstrating that phase synchronization can mitigate stiffness fluctuations.

(4) The coupling excitation amplitude *F*_ecc_ exhibits a prominent peak at Δ*ϕ* = π (*F*_couple_ ≈ 2300 N when *e*_1_ = 30 μm). The vibration risk is highest when the initial phase difference falls within the range of 90° to 270°, which coincides with the peak stiffness modulation region (Fig. 5c). This indicates that the stiffness-error coupling is the primary cause of vibration.

### Eccentricity ratio-phase synergistic mechanism

While the single-parameter analysis in Sect. 3.1 reveals the fundamental influence of phase difference on dynamic excitation, actual engineering applications involve coupled effects between multiple eccentricity parameters. To bridge the gap between isolated parameter analysis and real-world multi-parameter coupling, this section systematically investigates the synergistic effects between the eccentricity ratio (*e*_1_/*e*_2_) and the initial phase difference (Δ*ϕ*). This analysis aims to provide deeper insights into the nonlinear coupling mechanisms governing the system’s dynamic behavior under complex error conditions.

**Fig. 6:**
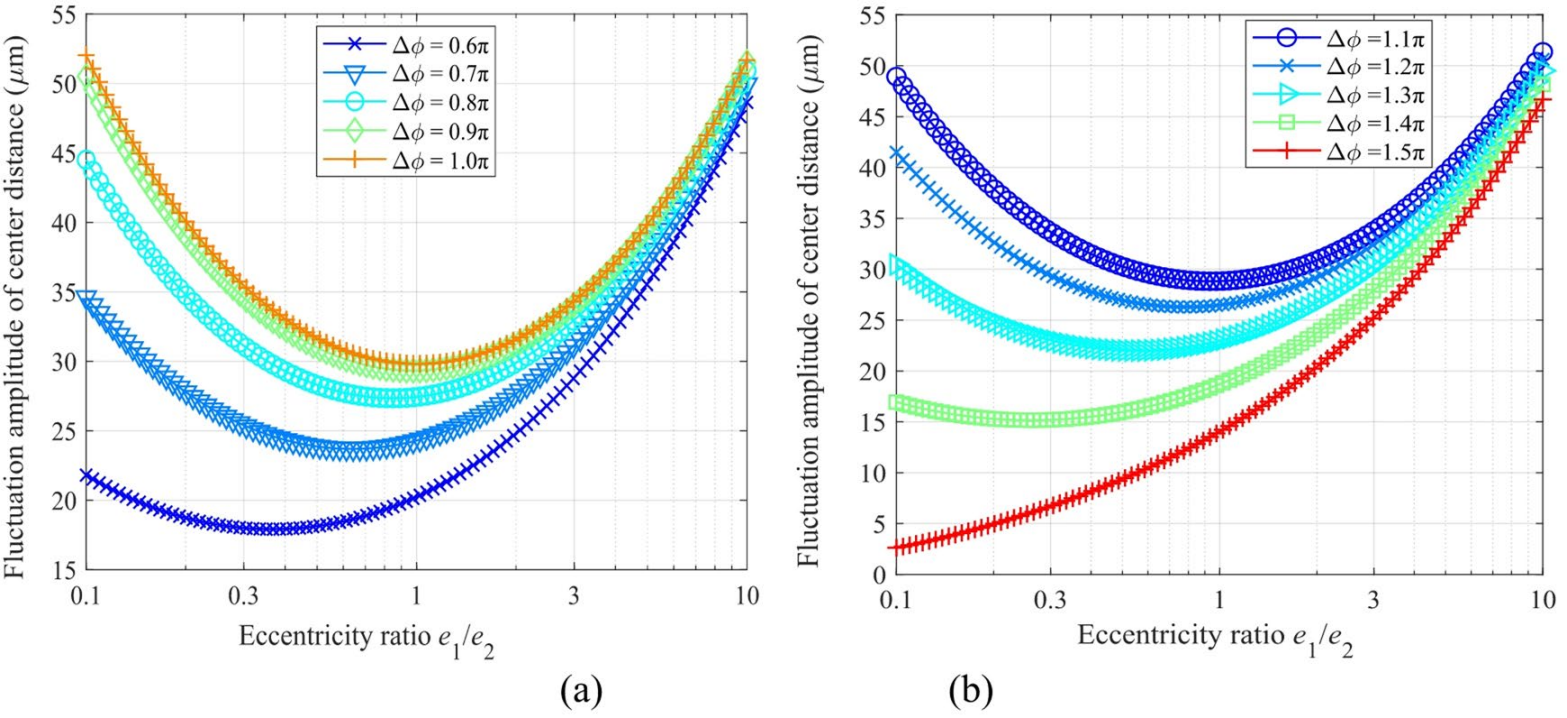
Variation of the center distance fluctuation amplitude with the eccentricity ratio (*e*_1_/*e*_2_ = 0.1 ~ 10): (**a**) initial phase difference range of (π/2, π), (**b**) initial phase difference range of (π, 3π/2).

Figure 6 shows the relationship between the amplitude of center distance fluctuation Δ*a* and the eccentricity ratio, where the eccentricity ratio *e*_1_/*e*_2_ varies from 0.1 to 10 (with the constraint that *e*_1_·*e*_2_ = (15 μm)², and the eccentric errors of *e*_3_ and *e*_4_ are not considered). Figure 6(a) corresponds to an initial phase difference range of (π/2, π), while Fig. 6(b) corresponds to a range of (π, 3π/2).

Figure 6 reveals that the center distance fluctuation amplitude Δ*a* follows a characteristic “U-shaped” distribution versus the eccentricity ratio *e*_1_/*e*_2_ for nearly all phase differences, suggesting an optimal configuration for minimizing vibrational fluctuations. For Δ*ϕ* ∈ (π/2, π) (Fig. 6a), the optimal ratio yielding the minimum Δ*a* falls between 0.5 and 1.0. Notably, the minimum amplitude at Δ*ϕ* = 0.6π (≈ 18 μm) is about 40% smaller than that at Δ*ϕ* = 1.0π (≈ 30 μm).

When Δ*ϕ* ∈ (π, 3π/2) (Fig. 6b), the optimization effect becomes even more pronounced. The global minimum fluctuation amplitude at Δ*ϕ* = 1.5π drops below 5 μm, representing a reduction of approximately 94% compared to the value at Δ*ϕ* = 1.1π. This phenomenon sheds light on the critical role of the initial phase difference in the motion compensation effect: an appropriate matching of the eccentricity ratio can achieve the optimal motion compensation. This finding provides a theoretical basis and design guidelines for parameter optimization in high-precision eccentric transmission systems.

**Fig. 7:**
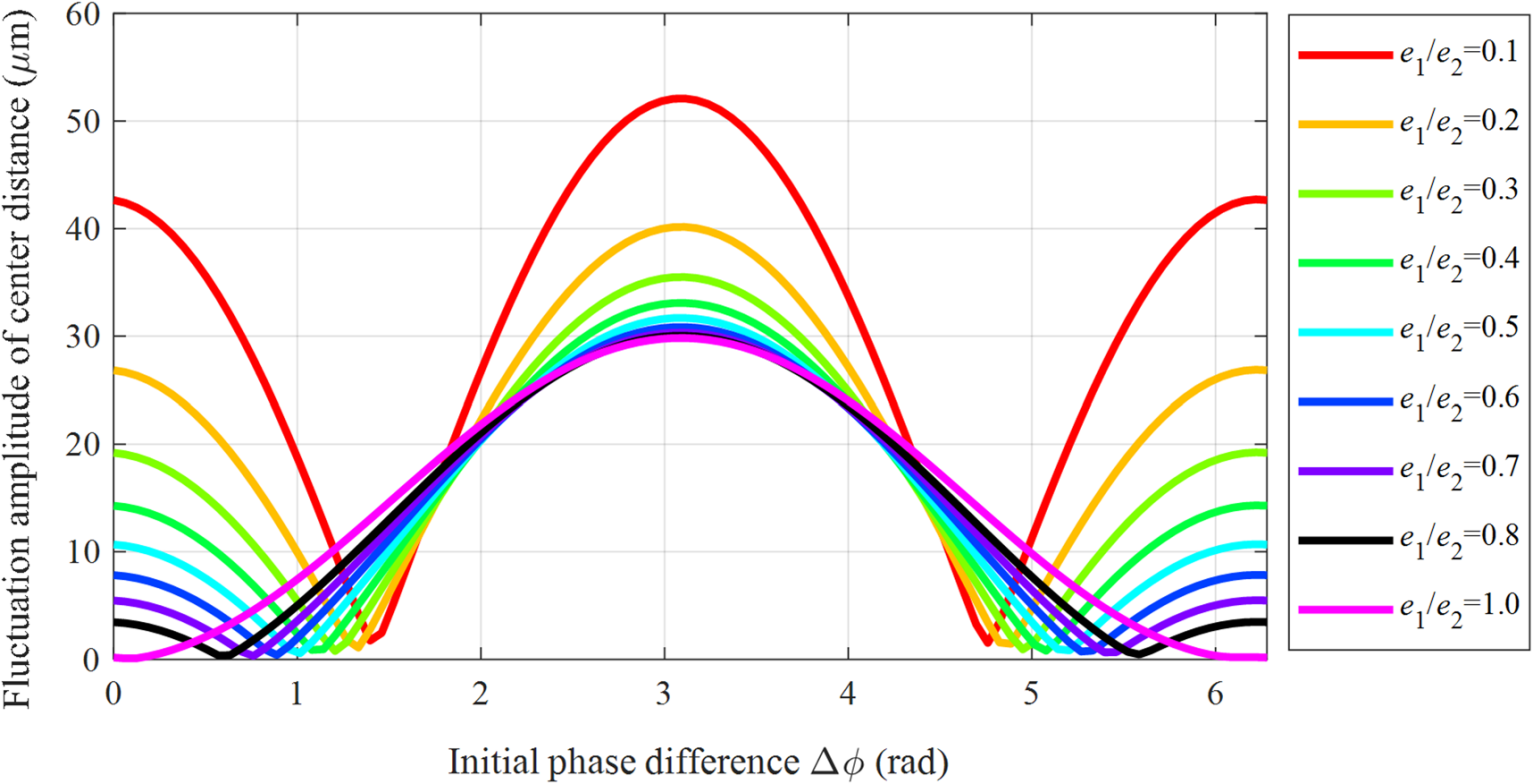
Relationship between center distance fluctuation amplitude Δ*a* and the initial phase difference Δ*ϕ*.

Figure 7 demonstrates the variation of the center distance fluctuation amplitude Δ*a* with the initial phase difference Δ*ϕ* for an eccentricity ratio *e*_1_/*e*_2_ ranging from 0.1 to 1 (eccentric errors *e*_3_ and *e*_4_ are neglected).

The results reveal a clear periodic variation in Δ*a* with a period of 2π. The extreme values of the amplitude are consistently located at phase differences equal to odd multiples of π (e.g., Δ*ϕ* = π, 3π, 5π). Results analysis reveals that the eccentricity ratio plays a decisive role in the fluctuation amplitude. At *e*_1_/*e*_2_ = 0.1, the maximum amplitude is 52 μm, whereas increasing the ratio to 1.0 substantially reduces it to about 30 μm — a 42.3% decrease. Moreover, a larger eccentricity ratio noticeably reduces both the curve oscillation amplitude and the phase sensitivity, demonstrating a significant improvement in system stability. This finding reveals that the center distance fluctuation can be effectively suppressed by rationally matching the eccentricity ratio with the phase difference, thereby providing a theoretical basis for the optimal design of high-precision gear.

**Fig. 8:**
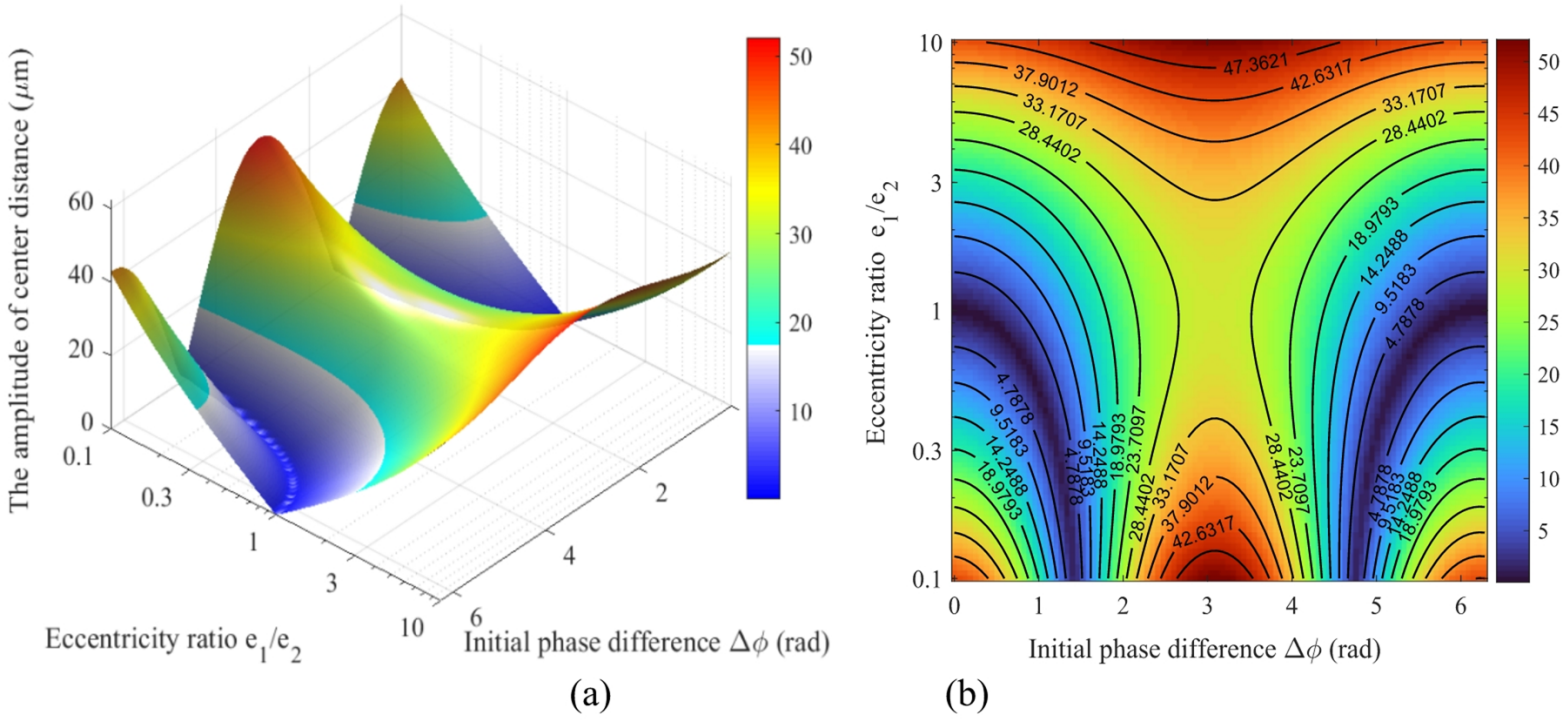
Relationship between the center distance fluctuation amplitude (Δ*a*) and the eccentricity ratio (*e*_1_/*e*_2_) and initial phase difference (Δ*ϕ*): (**a**) 3D surface map; (**b**) Heat map.

Figure 8 presents the fluctuation amplitude of the center distance Δa, as a function of the eccentricity ratio (*e*_1_/*e*_2_ = 0.1 ~ 10) and the initial phase difference (Δ*ϕ* = 0 ~ 2π). Figure 8(a) shows a 3D surface map, and Fig. 8(b) displays the corresponding heat map.

As shown in Fig. 8, the fluctuation amplitude of the center distance (Δa), exhibits a highly nonlinear coupling relationship with the eccentricity ratio (*e*_1_/*e*_2_) and the initial phase difference (Δ*ϕ*). The 3D surface map in Fig. 8(a) indicates that the system reaches its maximum amplitude (Δ*a*_max_ ≈ 52 μm) when Δ*ϕ* ≈ 0, π, or 2π, and *e*_1_/*e*_2_ approaches either 0.1 or 10. The heat map in Fig. 8(b) reveals two distinct “optimal valley” paths (dark blue regions in the heat map) within the parameter space. Specifically, when *e*_1_/*e*_2_ ≈ 1 and Δ*ϕ* ≈ 0 or 2π, Δ*a* can drop to a local minimum of approximately 5 μm. This phenomenon demonstrates the effectiveness of vibration suppression achieved through a phase cancellation mechanism. This relationship underscores that the cooperative optimization of both the eccentricity ratio and phase difference is essential for achieving high motion precision, as opposed to their independent selection.

**Fig. 9:**
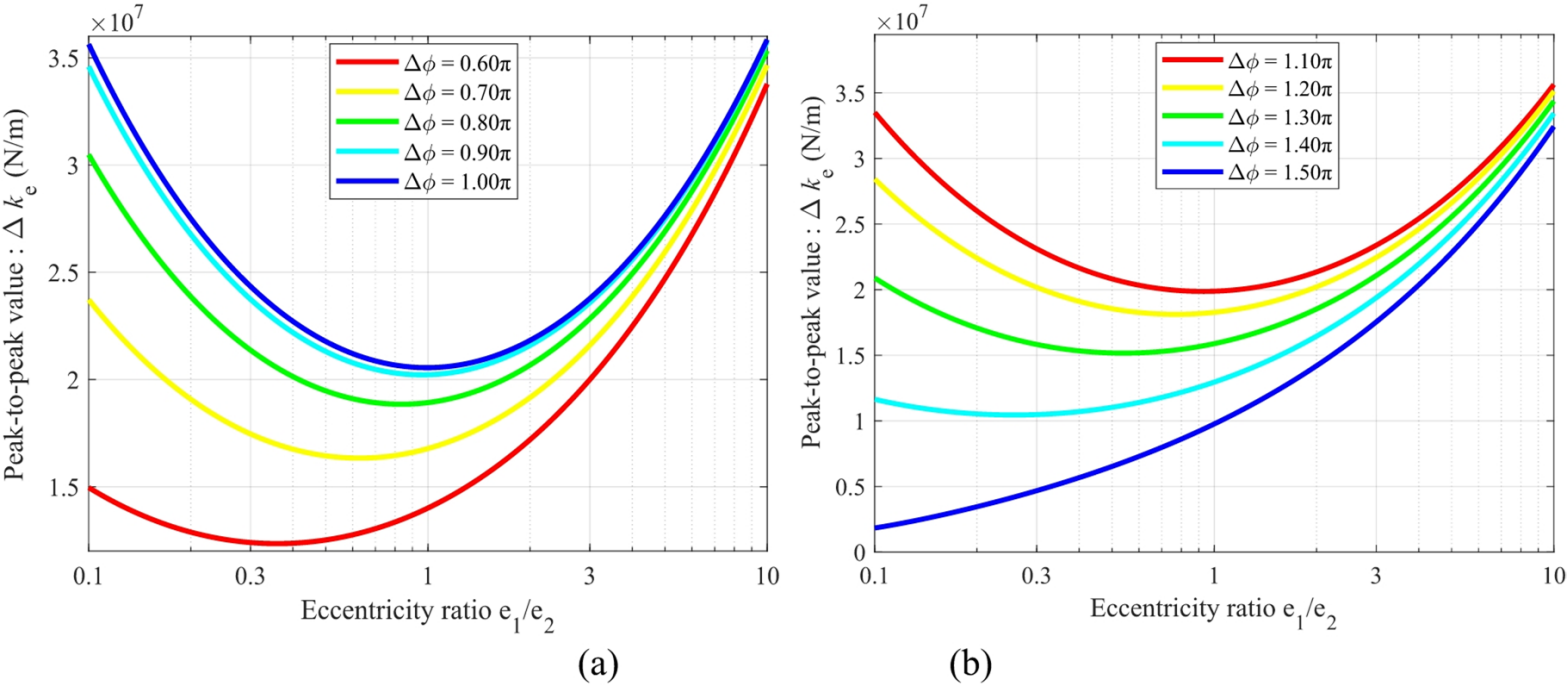
Mapping of the peak-to-peak modulated stiffness Δ*k*_*e*_ against the eccentricity ratio *e*_1_/*e*_2_ under different initial phase differences: (**a**) Δ*ϕ* ∈ (π/2, π), (**b**) Δϕ ∈ (π, 3π/2).

Figure 9 illustrates the relationship between the peak-to-peak value of the modulated stiffness Δ*k*_*e*_ versus the eccentricity ratio *e*_1_/*e*_2_ for different ranges of the initial phase difference Δ*ϕ*: (a) for Δ*ϕ* ∈ (π/2, π), and (b) for Δ*ϕ* ∈ (π, 3π/2).

Analysis of the results in Fig. 9 indicates a significant nonlinear coupling between Δ*k*_*e*_ and the eccentricity ratio *e*_1_/*e*_2_. Specifically, Δ*k*_*e*_ first decreases and then increases with the variation of *e*_1_/*e*_2_, reaching a local minimum near *e*_1_/*e*_2_ = 1 (e.g., Δ*k*_*e*min_ ≈ 2.0 × 10⁷ N/m at Δ*ϕ* = 0.9π). This reveals the fundamental role of a symmetric eccentric configuration (*e*_1_ = *e*_2_) in suppressing stiffness fluctuation. However, precise control of the phase difference remains a critical condition for achieving ultra-low stiffness variation.

**Fig. 10:**
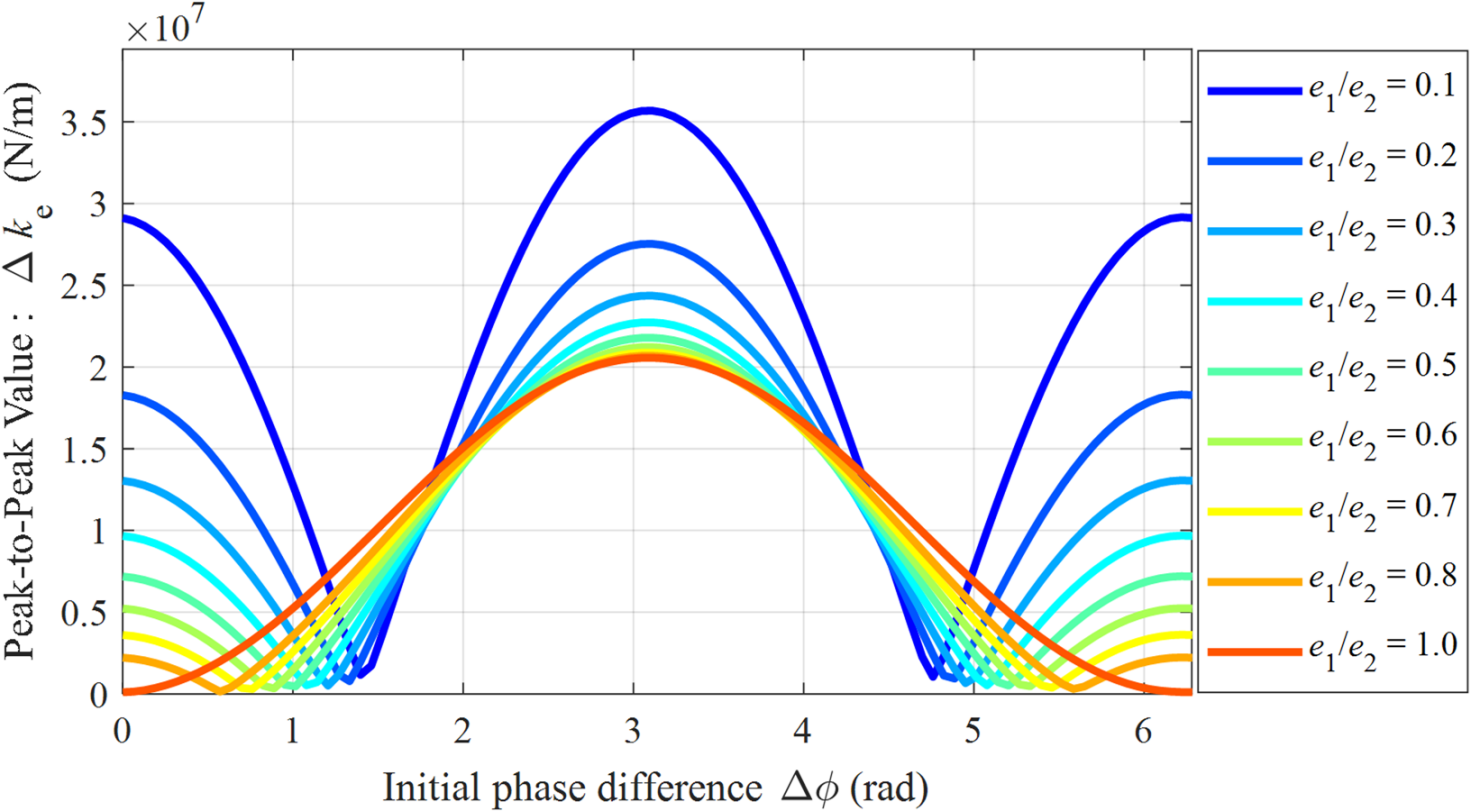
Mapping of the peak-to-peak modulated stiffness (Δ*k*_*e*_) versus the initial phase difference (Δ*ϕ*) under different eccentricity ratios.

Figure 10 illustrates the mapping relationship between the peak-to-peak value of the modulated stiffness (Δ*k*_*e*_) and the initial phase difference (Δ*ϕ*) under different eccentricity ratios (*e*_1_/*e*_2_). Analysis of the results in Fig. 10 indicates that Δ*k*_*e*_ is governed by a nonlinear coupling between Δ*ϕ* and *e*_1_/*e*_2_. For asymmetric eccentric structures (*e*_1_/*e*_2_ < 1), Δ*k*_*e*_ exhibits a distinct multi-peak characteristic as a function of Δ*ϕ*. The peaks occur near Δ*ϕ* ≈ *n*π (*n* = 0, 1, 2, …); for instance, Δ*k*_*e*max_ ≈ 3.5 × 10⁷ N/m when *e*_1_/*e*_2_ = 0.1. Conversely, the troughs are located near Δ*ϕ* ≈ π/2 and 3π/2. However, as the eccentricity ratio approaches 1, this multi-peak characteristic gradually diminishes and eventually evolves into a single-peak distribution centered at Δ*ϕ* = π. When *e*_1_/*e*_2_ = 1.0, the stiffness fluctuation amplitude is Δ*k*_*e*_ ≈ 2.0 × 10⁷ N/m, representing a 44.4% reduction compared to the maximum amplitude. This phenomenon confirms that a symmetric eccentric structure can effectively suppress stiffness fluctuation. Nevertheless, due to its inherent dynamic coupling characteristics that cannot be entirely eliminated, further system optimization requires controlling Δ*ϕ* away from π.

**Fig. 11:**
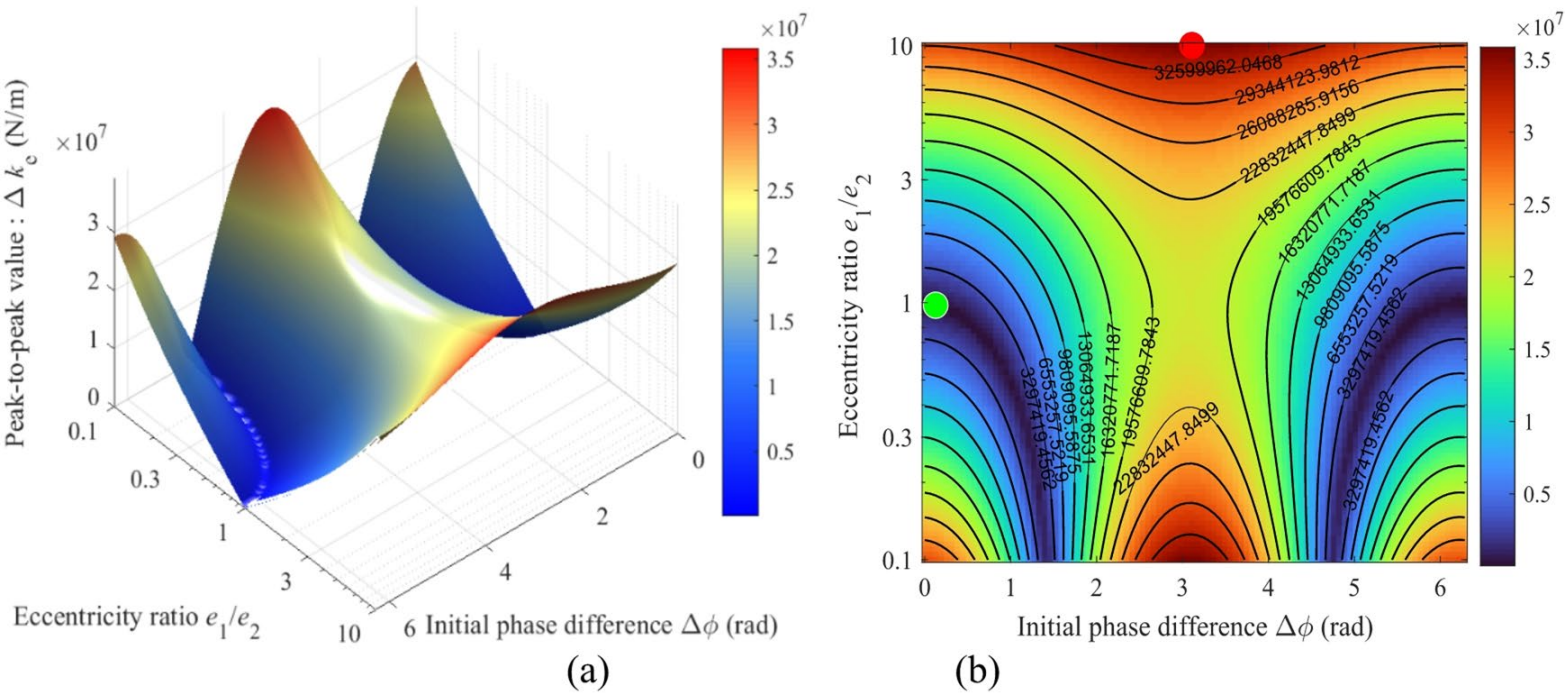
Relationship between the peak-to-peak modulated stiffness Δ*k*_*e*_ and the parameters *e*_1_/*e*_2_ and Δ*ϕ*: (**a**) 3D mapping; (**b**) Heat map.

The peak-to-peak modulated stiffness (Δ*k*_*e*_) exhibits a strong dependence on the nonlinear synergy between the eccentricity ratio (*e*_1_/*e*_2_) and the initial phase difference (Δ*ϕ*), as shown in Fig. 11. Both the 3D surface and the heat map demonstrate that the maximum system stiffness fluctuation (Δ*k*_*e*_ ≈ 3.5 × 10⁷ N/m) occurs under the combined condition of a highly asymmetric eccentricity ratio (*e*_1_/*e*_2_ = 0.1 or 10) and a specific phase difference (Δ*ϕ* = π), which is represented by the dark red regions indicating high values on the heat map.

It is noteworthy that a blue “canyon region”, centered at (*e*_1_/*e*_2_ = 1, Δ*ϕ* = 0 or 2π), exists within the parameter space. Here, Δ*k*_*e*_ can drop to the global minimum (< 0.5 × 10⁷ N/m). This finding provides a clear design pathway for vibration suppression: primarily adopting an equal-eccentricity design (*e*_1_/*e*_2_ ≈ 1) and strictly controlling the phase difference near 0 or 2π during assembly can inherently and significantly reduce the system’s stiffness modulation fluctuation.

**Fig. 12:**
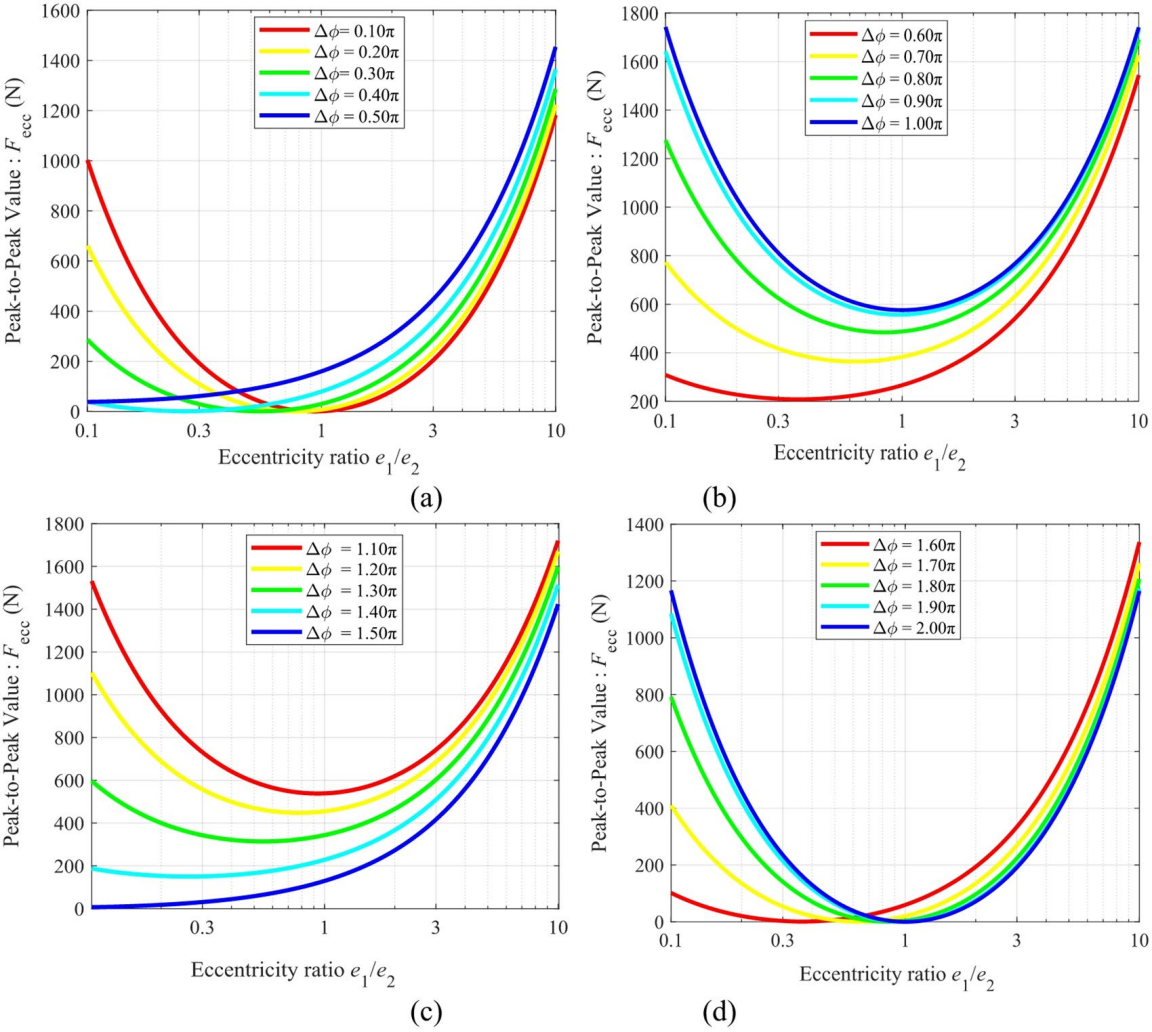
Mapping of the peak-to-peak eccentric excitation force (*F*_ecc_) against the eccentricity ratio for different ranges of Δ*ϕ*: (**a**) Δ*ϕ* ∈ (0, π/2), (**b**) Δ*ϕ* ∈ (π/2, π), (**c**) Δ*ϕ* ∈ (π, 3π/2), (**d**) Δ*ϕ* ∈ (3π/2, 2π).

Figure 12 shows the mapping of the peak-to-peak eccentric excitation force (*F*_ecc_) against the eccentricity ratio (*e*_1_/*e*_2_). A systematic analysis of Fig. 12 indicates that *F*_ecc_ is co-modulated by both the eccentricity ratio and the initial phase difference. All subfigures reveal that *F*_ecc_ exhibits a “U-shaped” distribution versus *e*_1_/*e*_2_, reaching its global minimum near the symmetric configuration (*e*_1_/*e*_2_ = 1). For example, the minimum value is approximately 0 N at Δ*ϕ* = 0.1π, and about 590 N at Δ*ϕ* = π. In contrast, *F*_ecc_ surges to its maximum under highly asymmetric conditions (*e*_1_/*e*_2_ = 0.1 or 10), reaching up to 1740 N when Δ*ϕ* = π. Therefore, to achieve an ultra-low eccentric excitation force (< 400 N), it is essential to adopt a symmetric eccentric structure (*e*_1_/*e*_2_ ≈ 1) and avoid the high-sensitivity region around Δ*ϕ* ≈ π.

**Fig. 13:**
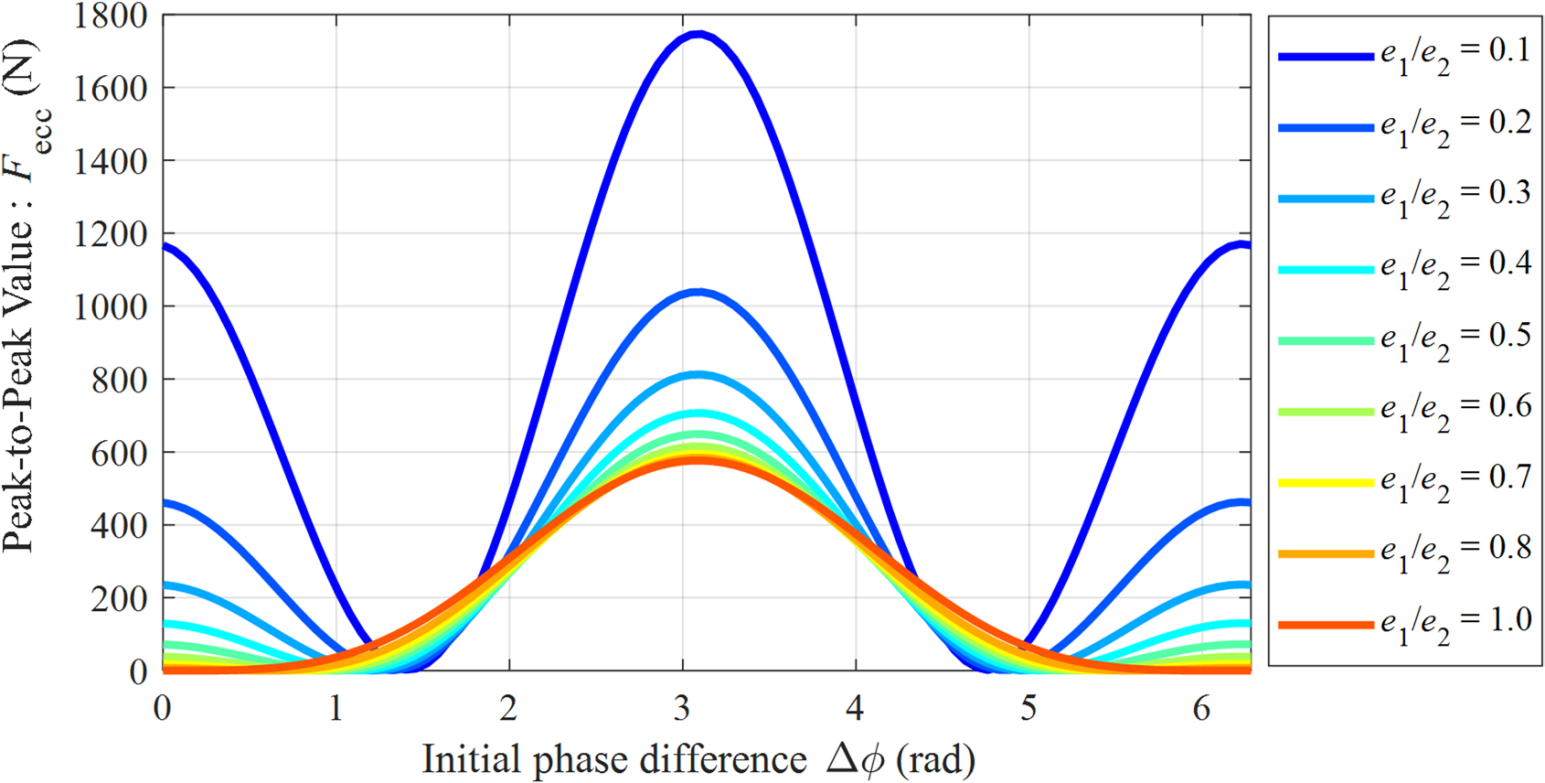
Mapping relationship between the peak-to-peak eccentric excitation force (*F*_ecc_) and the initial phase difference (Δ*ϕ*).

Figure 13 shows the mapping relationship between the peak-to-peak eccentric excitation force (*F*_ecc_) and the initial phase difference (Δ*ϕ*) under different eccentricity ratios. Analysis of Fig. 13 indicates that *F*_ecc_ reaches its global maximum at Δ*ϕ* = π for all eccentricity ratios considered, and the force amplitude increases with the degree of asymmetry. Specifically, under a highly asymmetric condition (*e*_1_/*e*_2_ = 0.1), the peak force reaches approximately 1740 N. As the eccentricity ratio approaches 1 (symmetric structure), the peak force decreases to a minimum of about 580 N, representing a significant reduction of 66.7%. This phenomenon clearly reveals that a symmetric eccentric design plays a fundamentally beneficial role in suppressing vibration excitation under severe operating conditions.

**Fig. 14:**
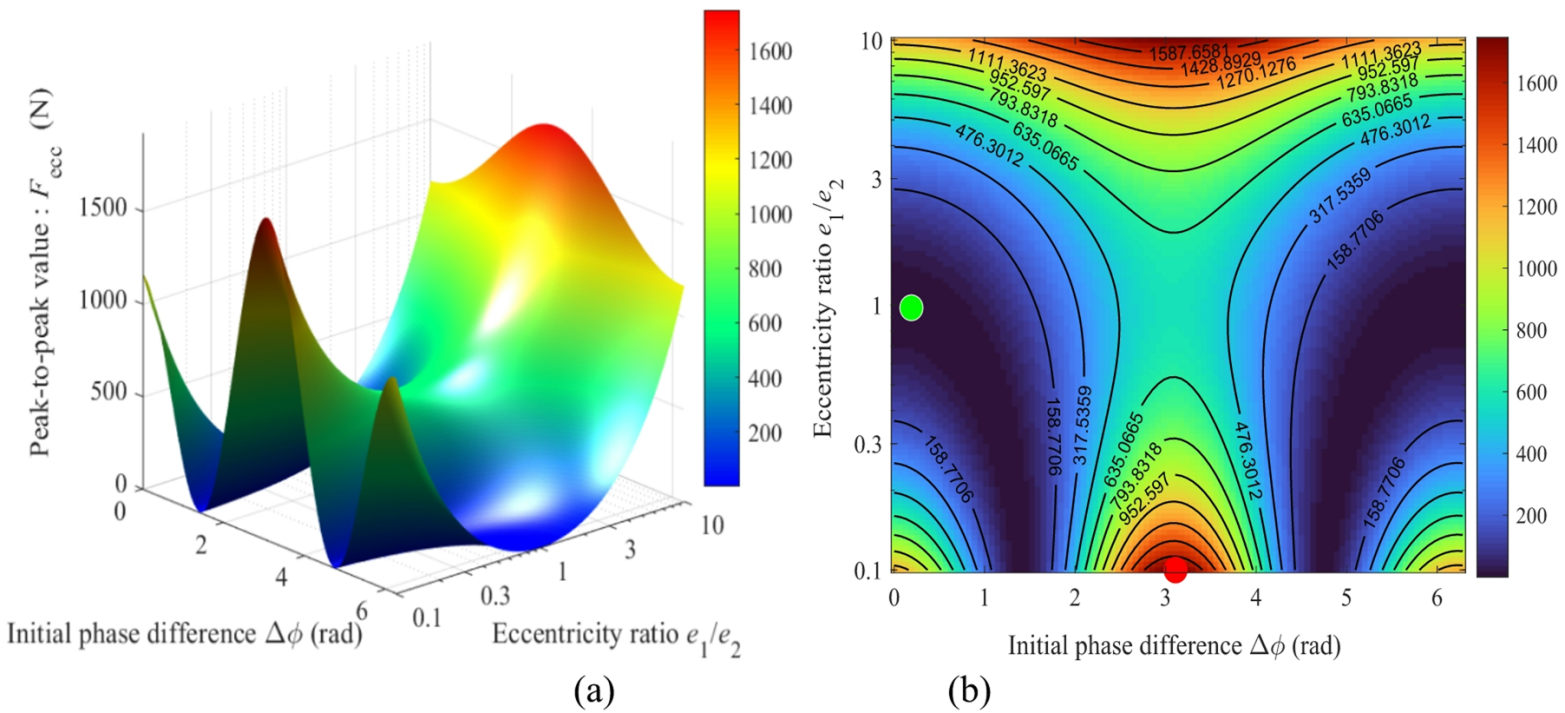
Relationship between the peak-to-peak eccentric excitation force *F*_ecc_ and the parameters *e*_1_/*e*_2_ and Δ*ϕ*: (**a**) 3D mapping; (**b**) Heat map.

Figure 14. shows the mapping of the peak-to-peak eccentric excitation force (*F*_ecc_) versus the eccentricity ratio (*e*_1_/*e*_2_) and the initial phase difference (Δ*ϕ*). The eccentricity ratio varies from 0.1 to 10, and the initial phase difference Δ*ϕ* ranges from 0 to 2π. Figure 14(a) shows the three-dimensional surface map, and Fig. 14(b) presents the heat map of the amplitude variation.

The 3D surface and heat map in Fig. 14 collectively demonstrate that the global maximum value (max{*F*_ecc_} = 1740 N) is located at the combination of a highly asymmetric eccentricity ratio (*e*_1_/*e*_2_ = 0.1 or 10) and a phase difference of Δ*ϕ* = π, which appears as the red high-value regions on both sides of the heat map. In stark contrast, the global minimum values are distributed within the dark blue symmetric regions of the heat map. Two specific examples are: first, under the symmetric eccentric condition (*e*_1_/*e*_2_ = 1 and Δϕ = 0 or 2π), and second, under a highly asymmetric but phase-orthogonal condition (*e*_1_/*e*_2_ = 0.1 and Δ*ϕ* = π/2 or 3π/2). This precise quantitative distribution proves that there are two effective yet distinct pathways for optimizing the excitation force: (1) If a symmetric eccentric design (*e*_1_/*e*_2_ = 1) is adopted, the phase difference must be strictly controlled near 0 or 2π; (2) If design constraints necessitate a highly asymmetric eccentric wheel (*e*_1_/*e*_2_ = 0.1), the phase difference should be maintained close to π/2 or 3π/2 to achieve optimal dynamic performance.

## Analysis of the dynamic response characteristics

To systematically investigate the influence of geometric eccentricity on the dynamic excitation of the MGTS, this section employs a translational-torsional coupled dynamic model based on the lumped parameter method (as shown in Fig. 3) to reveal the mapping relationship between eccentricity parameters and system response through numerical simulation. The Runge-Kutta method was used to numerically integrate the differential equations of motion (Eq. (1)), with a time step set to 10⁻⁵ s and a relative error tolerance of 10^− 4^, ensuring computational accuracy for high-frequency dynamic responses. The carrier output speed was 28.1 rpm, and the output load torque was 10 N⋅m.

### Influence of single eccentricity parameter

**Fig. 15:**
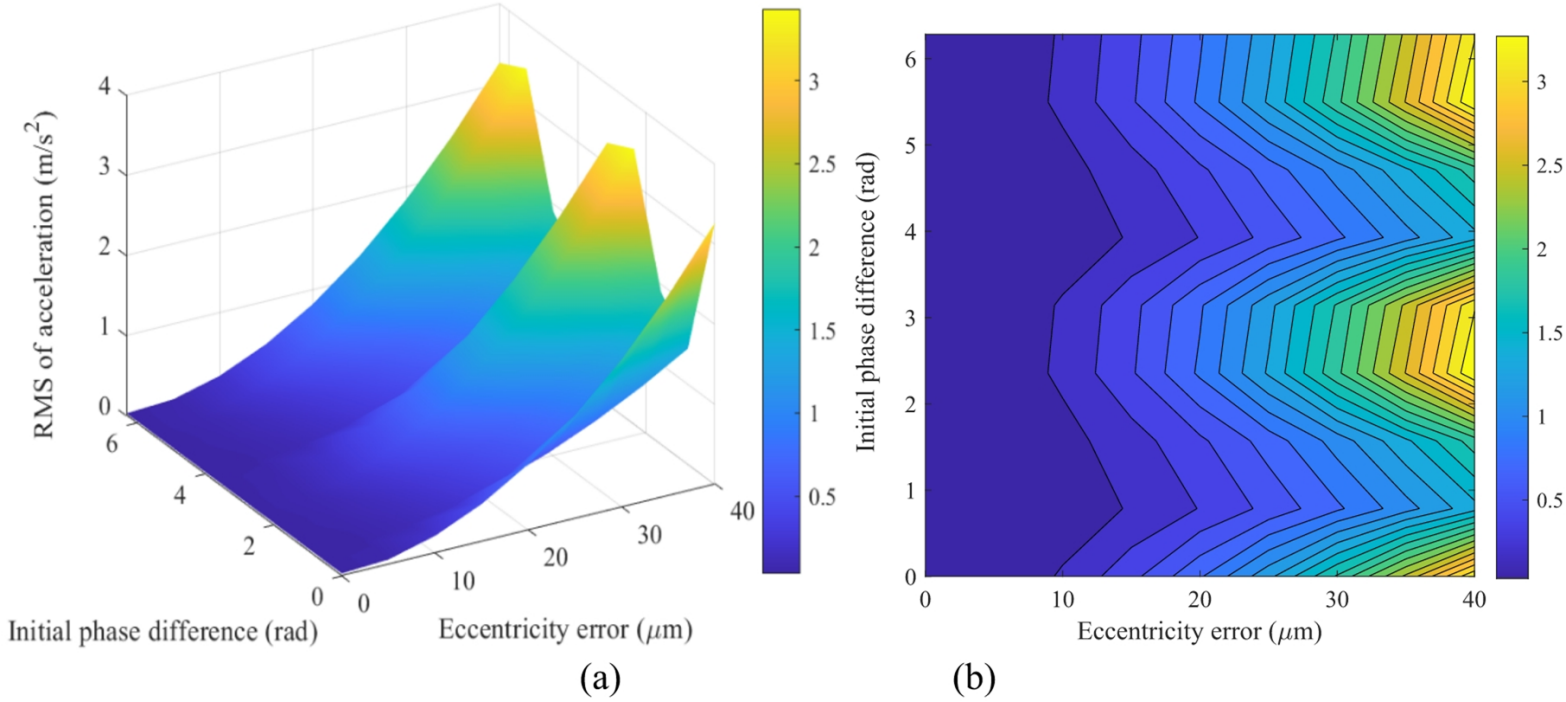
Dynamic response of the sun’s acceleration in the *x*-direction under different eccentricity errors and initial phase differences: (**a**) Three-dimensional mapping; (**b**) Amplitude contour map.

**Fig. 16:**
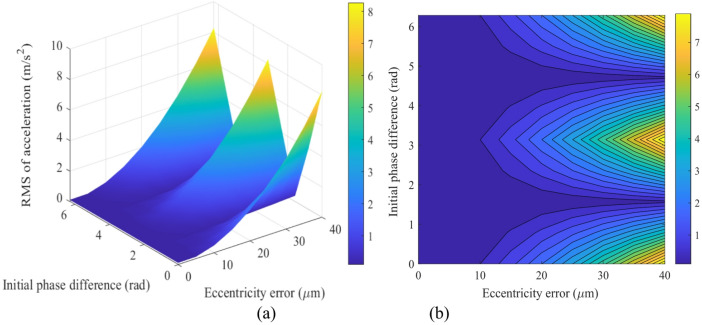
Dynamic response of the vibration acceleration of *g*_1_ in the *x*-direction under different eccentricity errors and initial phase differences: (**a**) Three-dimensional mapping; (**b**) Amplitude contour map.

The dynamic responses of the *x*-direction vibration acceleration are shown for the sun gear (Fig. 15) and gear *g*_1_ (Fig. 16) under various single eccentricity errors (*e*_1_) and initial phase differences (Δ*ϕ*).

A comprehensive analysis of the dynamic responses in Fig. 15 (sun gear) and Fig. 16 (gear *g*_1_) indicates that the system’s vibrational characteristics are governed by the coupled dynamics of the MGTS. The global maximum vibration acceleration of gear *g*_1_ is approximately 8 m/s², significantly higher than that of the sun gear (≈ 3.2 m/s²), revealing the attenuation of vibrational energy along the transmission path. Both components exhibit peak responses under the combination of a large eccentricity error (*e*_1_ = 40 μm) and a specific phase difference (Δ*ϕ* ≈ 3 ~ 4 rad, i.e., near π), identifying this parameter region as a high-risk zone for vibration. In contrast, the low vibration region (< 1 m/s²) is concentrated within the “safe zone” of small eccentricity errors (*e*_1_ < 10 μm). This pattern conclusively demonstrates that suppressing system vibration hinges on controlling gear manufacturing and assembly precision while synergistically optimizing the assembly phase. This strategy avoids the amplification of excitation from the preceding stages and thereby globally enhances the dynamic performance of the transmission chain.

**Fig. 17:**
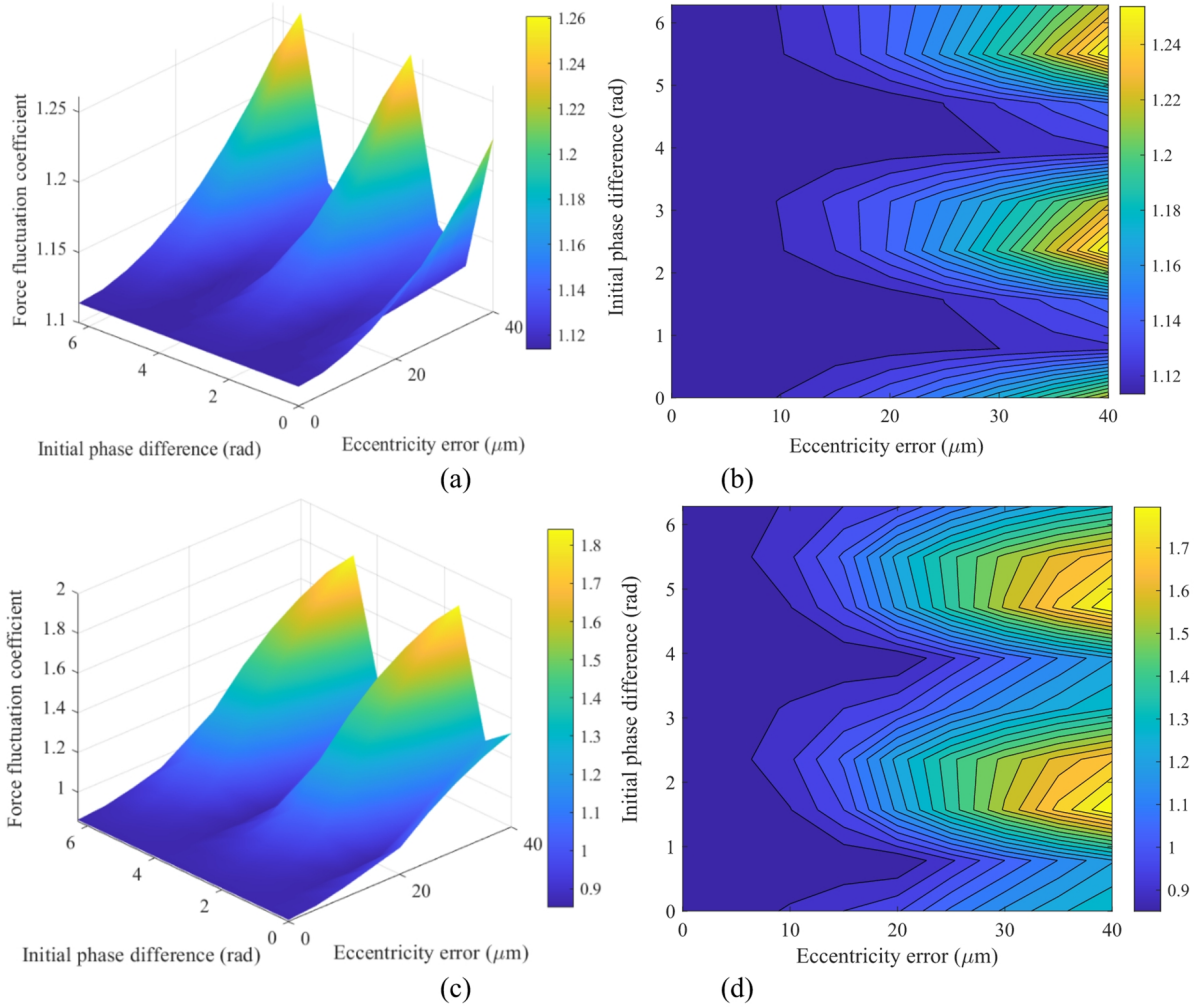
3D mapping of the dynamic meshing force fluctuation coefficient *L*_*f*_ versus eccentricity *e*_1_ and phase difference Δ*ϕ*: (**a**) (**b**) for force *F*_*sp*1_; (**c**)(**d**) for force *F*_*sp*3_.

Figure 17 illustrates the 3D mapping relationship between the fluctuation coefficient (*L*_*f*_ = (*F*_max_-*F*_min_)/*F*_mean_) of the dynamic meshing force in the PGS and both the gear eccentricity (*e*_1_) and the initial phase difference (Δ*ϕ*) between *e*_1_ and *e*_2_. Figure 17 reveals the fluctuation characteristics of the dynamic meshing forces for different gear pairs within the PGS: the fluctuation coefficient amplitude of *F*_*sp*1_ is relatively low (ranging from 1.12 to 1.25), with its high-risk zone concentrated in the combination of large eccentricity errors (*e*_1_ > 30 μm) and a specific phase range (Δ*ϕ* ≈ 3 ~ 4 rad). In contrast, the load fluctuation of *F*_*sp*3_ is more pronounced (ranging from 0.9 to 1.8), exhibiting not only a wider amplitude range but also greater sensitivity to parameter variations. This stark contrast indicates that eccentric errors disrupt the inherent load-sharing characteristics among the planetary gears, leading to a significant redistribution and intense fluctuation of the load among them.

### Analysis of multi-parameter coupling effects

**Fig. 18:**
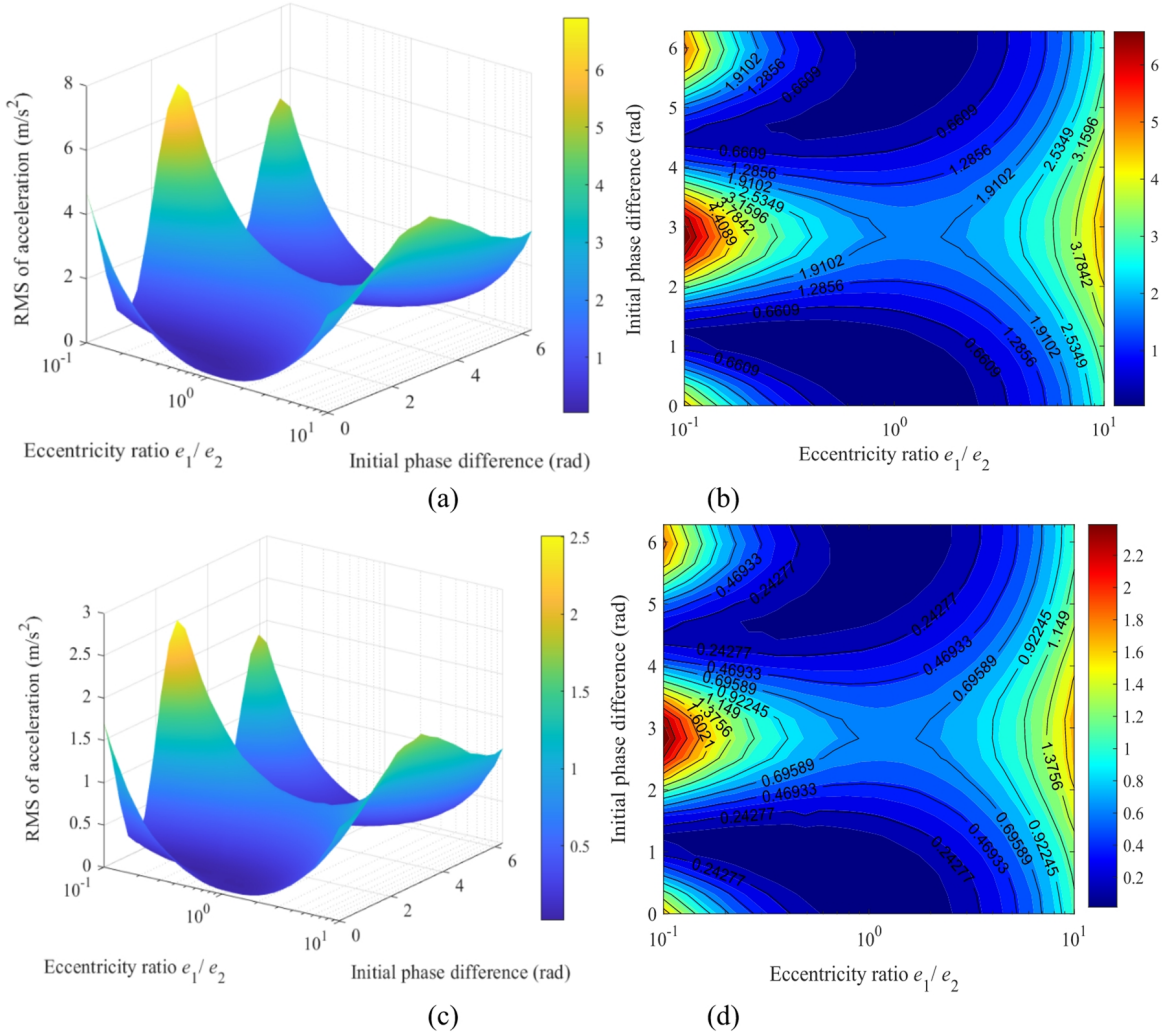
Mapping of the sun’s acceleration responses versus the eccentricity ratio (*e*_1_/*e*_2_) and initial phase difference (Δ*ϕ*): (**a**) 3D surface map of the *x*-direction acceleration; (**b**) Heat map of the *x*-direction acceleration; (**c**) 3D surface map of the *y*-direction acceleration; (**d**) Heat map of the *y*-direction acceleration.

Figure 18 illustrates the mapping relationships between the acceleration responses of the sun gear in the x- and y-directions relative to the eccentricity ratio (*e*_1_/*e*_2_) and the initial phase difference (Δ*ϕ*). The results indicate that the global maximum vibration responses of the sun gear (approximately 6.8 m/s² in the *x*-direction and 2.5 m/s² in the *y*-direction) consistently occur within the same high-risk region of the parameter space. This region corresponds to a combination of an eccentricity ratio of *e*_1_/*e*_2_ ≈ 0.1 and an initial phase difference of Δ*ϕ* ≈ π, appearing as steep peaks in the 3D surface plots and the most prominent red high-value areas in the heatmaps. This phenomenon demonstrates that the combination of a highly asymmetric eccentricity configuration and an antiphase operating condition excites the most severe dynamic unbalanced excitation, leading to intense vibrations of the sun gear in both translational directions. In contrast, the most stable low-vibration state globally is observed in another region of the parameter space: operating conditions with a symmetric or near-symmetric eccentric structure (*e*_1_/*e*_2_ ≈ 1) and a phase difference far from Δ*ϕ* = π, which manifest as blue low-value regions in the figures.

**Fig. 19:**
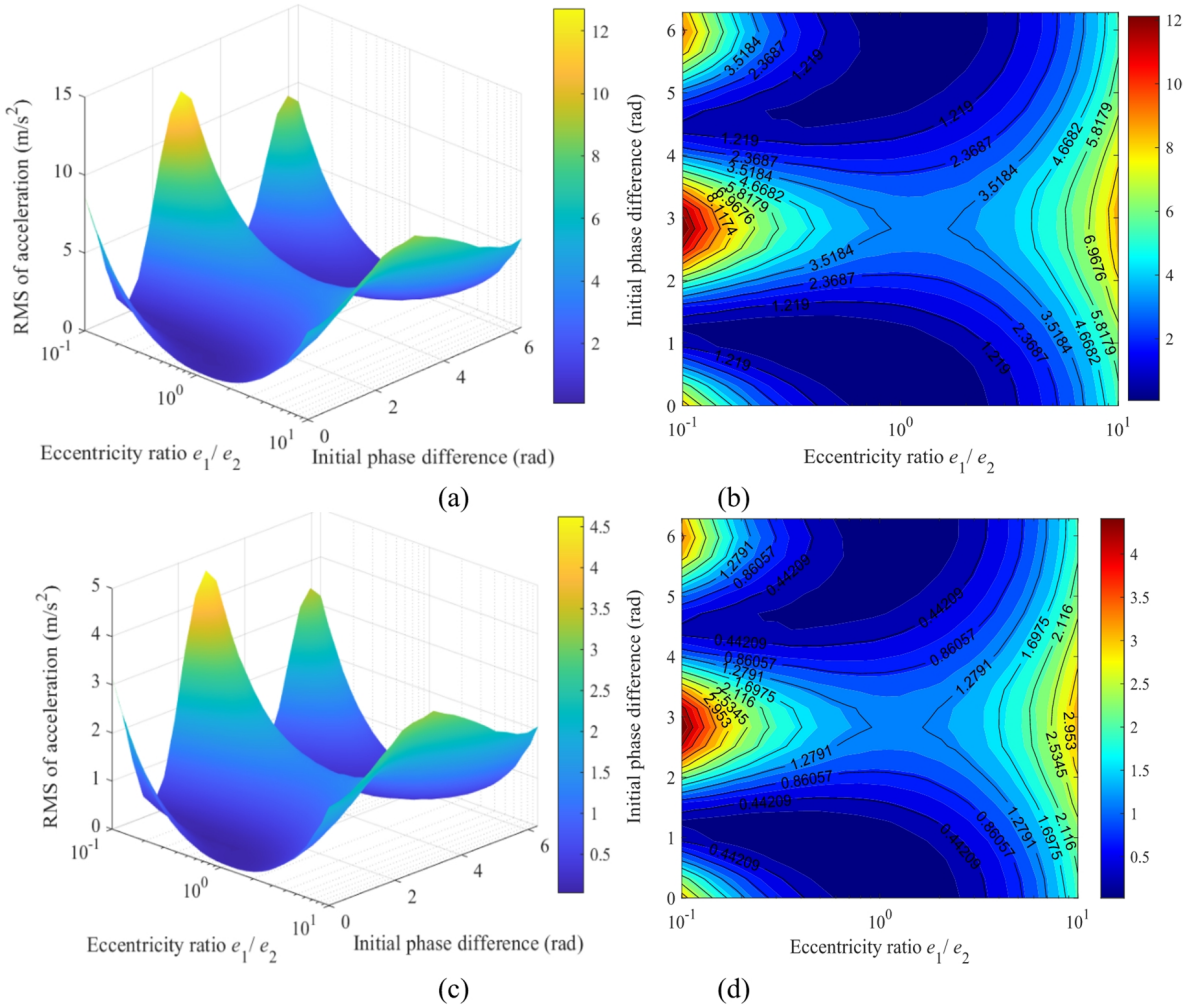
Mapping of the acceleration responses of gear *g*_4_ versus the eccentricity ratio (*e*_1_/*e*_2_) and initial phase difference (Δ*ϕ*): (**a**) 3D surface map of the *x*-direction acceleration; (**b**) Heat map of the *x*-direction acceleration; (**c**) 3D surface map of the *y*-direction acceleration; (**d**) Heat map of the *y*-direction acceleration.

Figure 19 illustrates the mapping relationship between the acceleration responses of gear *g*_4_ and the eccentricity ratio (*e*_1_/*e*_2_) as well as initial phase difference (Δ*ϕ*). The global maximum vibration response occurs precisely at the same high-risk parameter combination: a highly asymmetric eccentricity ratio (*e*_1_/*e*_2_ ≈ 0.1) under an anti-phase condition (Δ*ϕ* ≈ π). Under this condition, the root mean square (RMS) of acceleration reaches approximately 12 m/s² in the *x*-direction (Fig. 19a, b) and about 4.5 m/s² in the *y*-direction (Fig. 19c, d). These amplitudes are significantly higher than the responses of the sun gear under identical conditions, indicating that the high-speed stage acts as the primary excitation source and the key amplification stage for system vibrations.

In summary, the mapping relationships in Fig.s 18 and 19 provide clear design guidelines for vibration control of the transmission system: the high-risk parameter combination of “asymmetry + anti-phase” (*e*_1_/*e*_2_ < < 1 and Δ*ϕ* = π) must be avoided. To achieve high performance, a symmetric eccentric design (*e*_1_/*e*_2_ ≈ 1) should be prioritized, with the phase difference set near Δ*ϕ* ≈ π/2 or 3π/2, thereby suppressing vibration at the source.

**Fig. 20:**
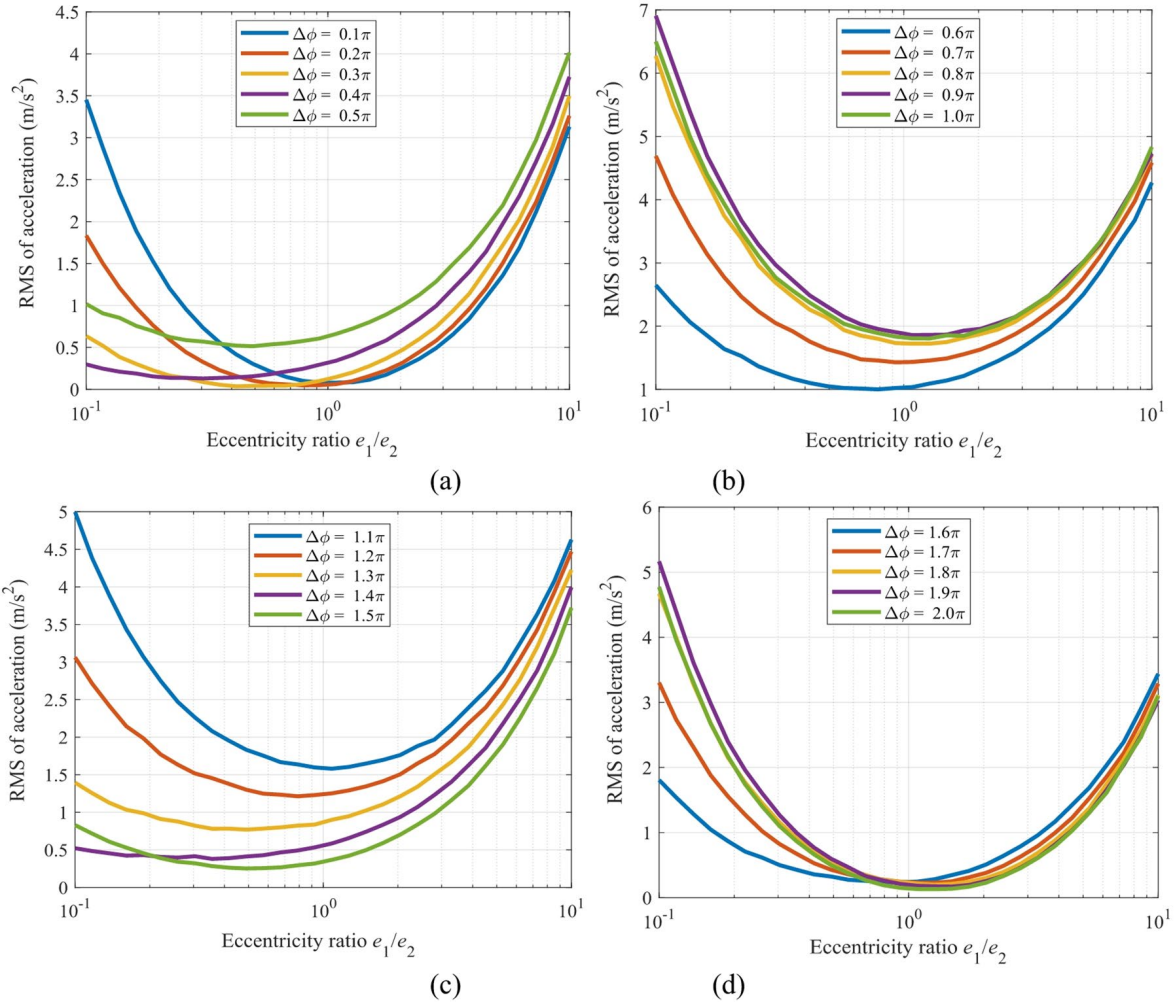
Mapping of *x*-direction acceleration of the sun versus the eccentricity ratio (*e*_1_/*e*_2_): (**a**) Δϕ in the range of (0, π/2); (**b**) Δ*ϕ* in (π/2, π); (**c**) Δ*ϕ* in (π, 3π/2); (**d**) Δ*ϕ* in (3π/2, 2π).

**Fig. 21:**
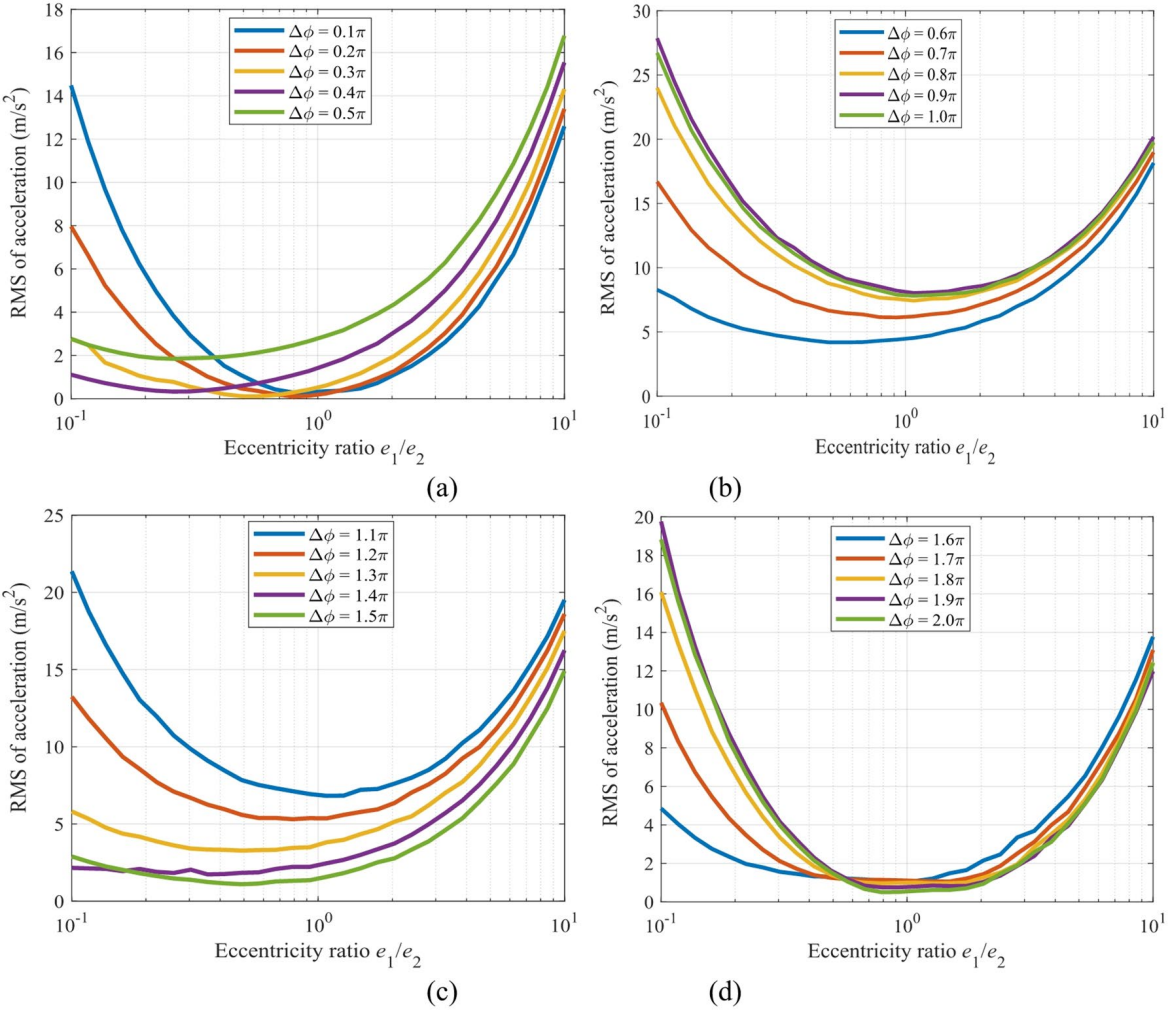
Mapping of *x*-direction acceleration of the gear *g*_2_ versus the eccentricity ratio (*e*_1_/*e*_2_): (**a**) Δ*ϕ* in the range of (0, π/2); (**b**) Δ*ϕ* in (π/2, π); (**c**) Δ*ϕ* in (π, 3π/2); (**d**) Δ*ϕ* in (3π/2, 2π).

The mapping relationships between the RMS value of the *x*-direction acceleration and the eccentricity ratio (*e*_1_/*e*_2_) are shown in Fig. 20 for the sun gear and Fig. 21 for the gear *g*_2_.

Figure 20 reveals a strong nonlinear dependence between the vibration acceleration response of the sun gear and both the eccentricity ratio (*e*_1_/*e*_2_) and the initial phase difference (Δ*ϕ*). A comprehensive view of the four subfigures shows that all curves exhibit a significant “U-shaped” distribution. Specifically, the vibration acceleration reaches its global minimum near the symmetric structure (*e*_1_/*e*_2_ ≈ 1), with values as low as < 1.0 m/s², while it increases sharply to a peak (up to 7.0 m/s²) under highly asymmetric configurations (*e*_1_/*e*_2_ → 0.1 or 10). This confirms the fundamental role of a symmetric eccentric design in vibration suppression. The phase difference Δ*ϕ* is a key factor modulating the amplitude of this “U-shaped” curve: for a fixed eccentricity ratio, the curve’s amplitude shows an increasing trend as Δ*ϕ* approaches π, reaching its global maximum precisely at Δ*ϕ* = π.

The mapping relationship between Figs. 20 and 21 provides clear design criteria for vibration control: To achieve the optimal dynamic performance, symmetrical or nearly symmetrical eccentric wheel designs (*e*_1_/*e*_2_ ≈ 1) should be given priority, and the assembly phase difference should be set as close as possible to Δ*ϕ* ≈ π/2 or 3π/2, so as to simultaneously utilize the inertial balance effect of the symmetrical structure and the excitation cancellation effect of the phase difference, thereby essentially suppressing vibration.

**Fig. 22:**
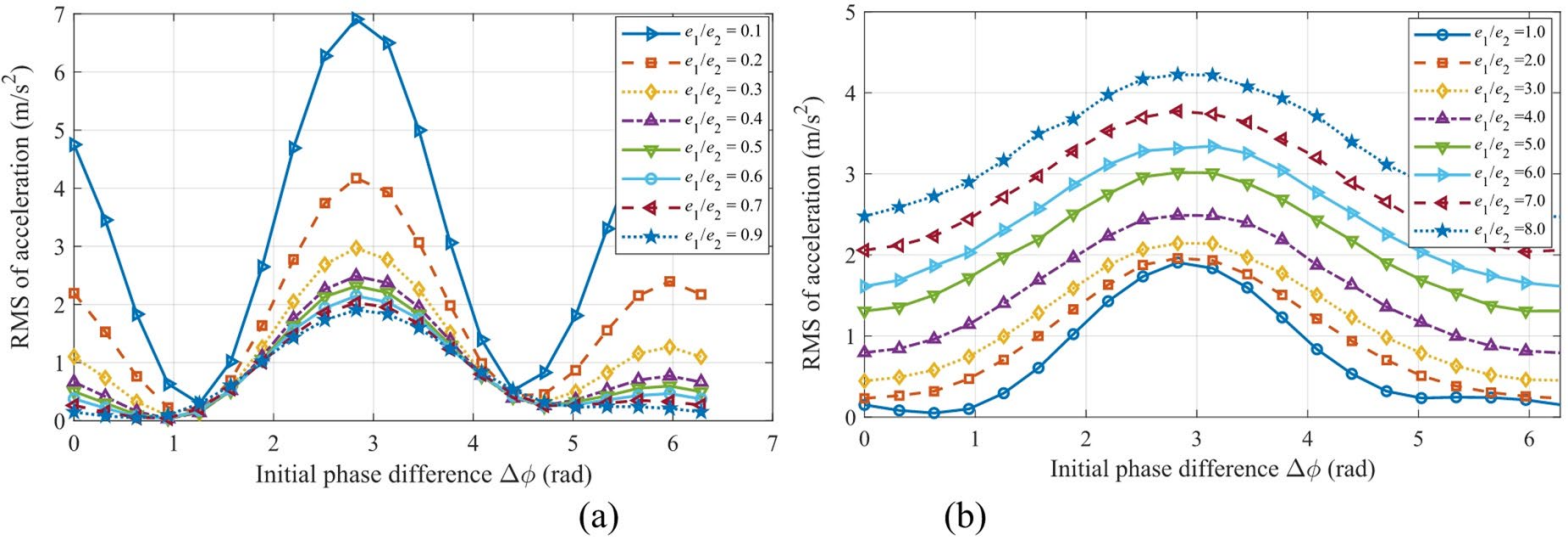
Mapping of the vibration acceleration for the sun gear in the *x*-direction versus the initial phase difference Δ*ϕ*: (**a**) For eccentricity ratio *e*_1_/*e*_2_ < 1; (**b**) For eccentricity ratio *e*_1_/*e*_2_ > 1.

Figure 22 shows the mapping relationship between the peak acceleration of the sun gear in the *x*-direction and the initial phase difference Δ*ϕ* under different eccentricity ratios. Specifically, Fig. 22(a) presents the curves for eccentricity ratios less than 1, while Fig. 22(b) displays the curves for eccentricity ratios greater than 1. A comprehensive analysis of both subfigures reveals that the response curves under all eccentricity ratios exhibit a periodic distribution symmetric about Δ*ϕ* = π (≈ 3.14 rad), reaching their global maxima near this point. For instance, the peak value approaches 7.0 m/s² when *e*_1_/*e*_2_ = 0.1, and reaches 4.2 m/s² when e₁/e₂ = 8.0.

**Fig. 23:**
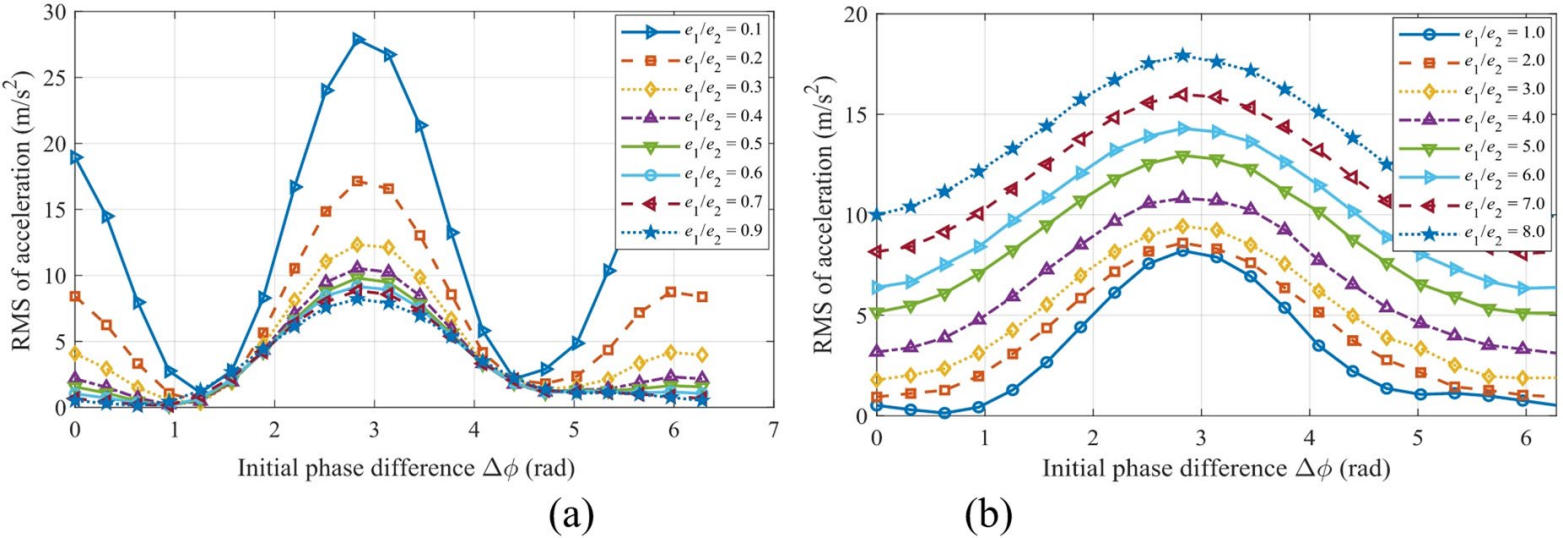
Mapping of the vibration acceleration of gear *g*_2_ in the *x*-direction versus the initial phase difference Δ*ϕ*: (**a**) For eccentricity ratio *e*_1_/*e*_2_ < 1; (**b**) For eccentricity ratio *e*_1_/*e*_2_ > 1.

Figure 23 illustrates the mapping relationship between the peak vibration acceleration of gear *g*_2_ in the *x*-direction and the initial phase difference Δ*ϕ* under various eccentricity ratios. A comprehensive analysis of Fig.s 22 and 23 reveals that the optimal phase difference is strongly dependent on the specific value of the eccentricity ratio. When *e*_1_/*e*_2_ < 1 (Fig. 22a), the low-vibration regions are concentrated near Δ*ϕ* ≈ π/2 and 3π/2. Conversely, when *e*_1_/*e*_2_ > 1 (Fig. 22b), these low-vibration regions shift to the vicinity of Δ*ϕ* ≈ 0 and 2π. This critical finding demonstrates that the optimal setting of the phase difference must be adjusted according to the specific eccentricity ratio configuration: quadrature phases (Δ*ϕ* = π/2, 3π/2) should be selected for small eccentricity ratios, while in-phase conditions (Δ*ϕ* = 0, 2π) are preferable for large eccentricity ratios. This finding provides a theoretical basis for the phase optimization of precision transmission systems.

### Engineering application: toward an optimized structural design

**Fig. 24:**
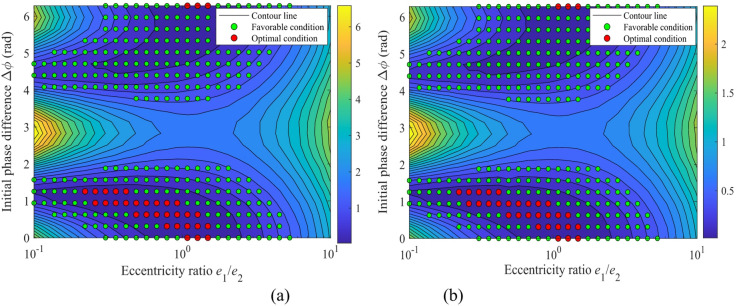
Contour maps of vibration acceleration in different directions of the sun gear with optimal operating regions: (**a**) For the *x*-direction; (**b**) For the *y*-direction.

**Fig. 25:**
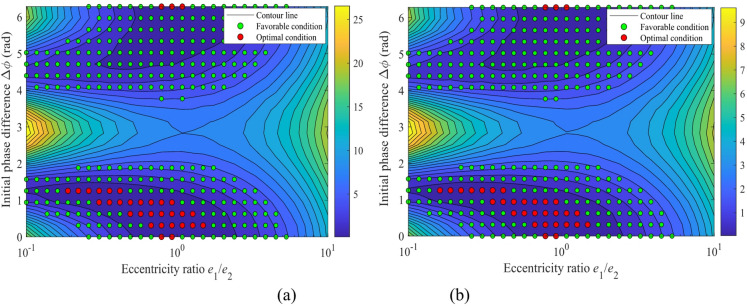
Contour maps of vibration acceleration in different directions of the gear *g*_2_ with optimal operating regions: (**a**) For the *x*-direction; (**b**) For the *y*-direction.

Figure 24 shows the contour map of the vibration acceleration of the sun gear in the *x* and *y* directions, while Fig. 25 shows the corresponding contour map for gear *g*_2_. The green dotted area represents the favorable operating condition, and the red dotted area indicates the optimal operating condition. Through a parameter scan of the system’s vibration response, Figs. 24 and 25 clearly delineate the optimization design domain based on dynamic performance: the red dotted area (optimal condition) corresponds to a narrow parameter space with an eccentricity ratio of *e*_1_/*e*_2_ ≈ 0.5 ~ 2 and an initial phase difference of Δ*ϕ* ≈ 0 ~ 1.5 rad. This combination reduces the system vibration level to the global minimum, providing a clear and critical design target for precision transmission systems with stringent vibration suppression requirements. The green dotted area (favorable condition) covers a wider range of eccentricity ratios, forming a robust design window that is less sensitive to parameter variations and offers greater robustness. Comprehensive analysis indicates that for performance optimization, parameters should be prioritized within the red optimal zone; if engineering tolerance and cost-effectiveness are considered, the extensive green zone provides a reliable and flexible design alternative.

## Experimental validation

### Construction of the three-stage transmission test rig

**Fig. 26:**
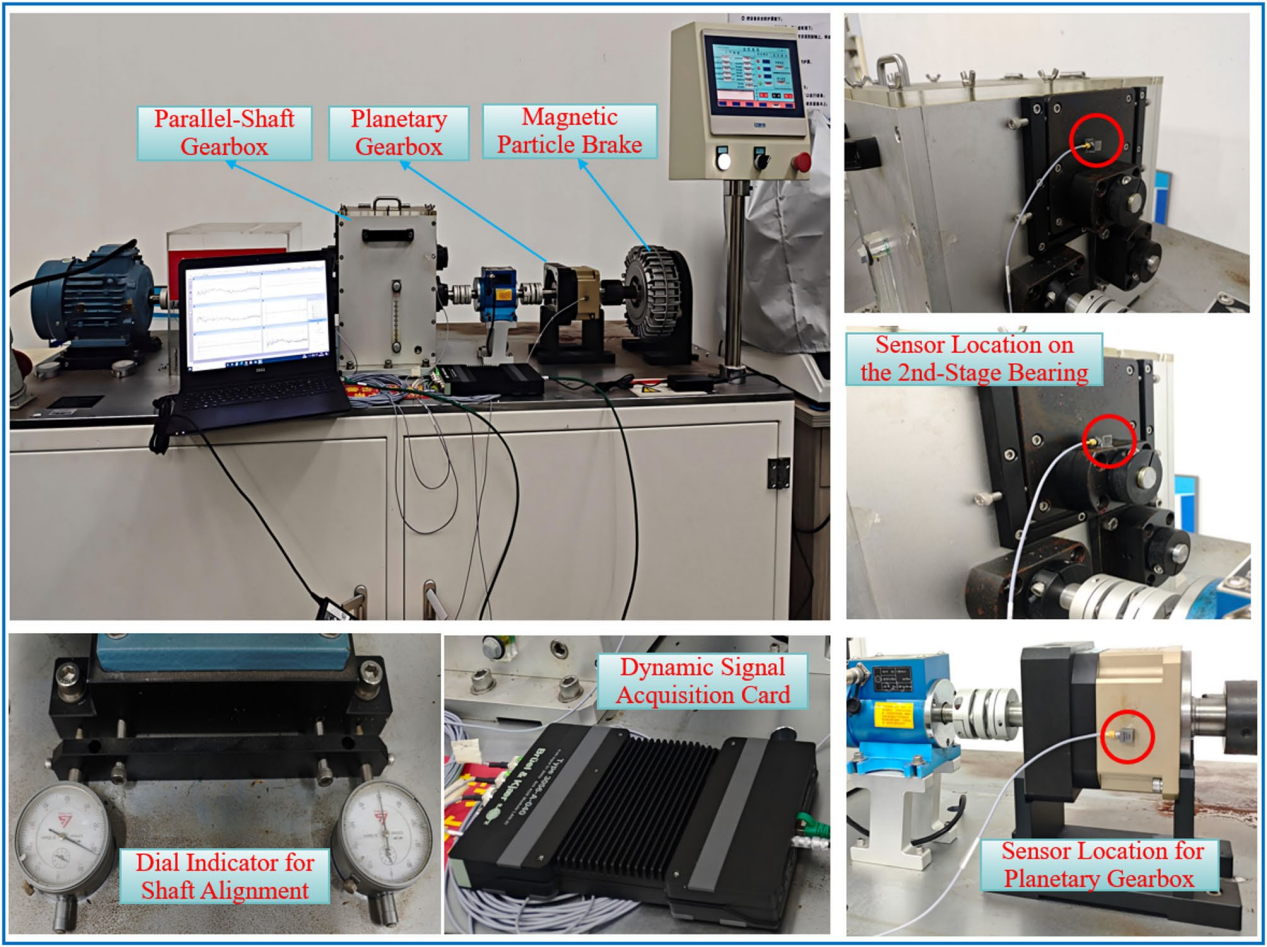
Three-stage transmission system test rig.

To validate the aforementioned theoretical model and the results of the dynamic characteristic analysis, this study established a three-stage transmission system test rig, as shown in Fig. 26. The platform is composed of a drive motor, a two-stage fixed-axis gearbox, a one-stage PGS, and a magnetic particle brake connected in series, thereby simulating a composite transmission path that includes both high-speed and low-speed stages. A precise load torque is applied to the system output by the magnetic particle brake. The load for this experimental condition was set to *T*_out_ = 10 N⋅m, consistent with the simulation settings.

The load application scheme is as follows: A precise load torque is applied to the system output via a magnetic particle brake. To avoid impact transients due to instantaneous loading and to simulate actual operating conditions, the load is applied gradually. It is increased smoothly from 0 N⋅m to the target value of *T*_out_ = 10 N⋅m at a rate of 2 N⋅m/s, a setting consistent with the simulation conditions. After system startup and load application, operation is maintained until a steady state is confirmed. This confirmation is based on monitoring the stability of the vibration signals’ time-domain waveforms and frequency spectra, a process typically requiring 3–5 min. This procedure ensures the system has exited the transient state. To comprehensively evaluate the dynamic characteristics of the system under different operating states, tests were conducted at three input speeds: 200 rpm, 400 rpm, and 800 rpm.

To ensure the reliability of the experimental data, the steady-state vibration signals were acquired for a duration of no less than five minutes under each input rotational speed condition (200 rpm, 400 rpm, and 800 rpm) with the load torque fixed at 10 N⋅m. The system was allowed to run for a sufficient period prior to data acquisition to confirm it had reached a steady-state operation, which was verified by monitoring the stability of the time-domain waveform and frequency spectrum of the vibration signal. For a consistent and representative analysis, a stable 60-second segment from the steady-state portion of the data was extracted for detailed signal processing and comparison with the simulation results. This approach effectively avoided transients during start-up and ensured signal stability for the comparative analysis presented in the following section.

Vibration responses were measured using a Brüel & Kjær data acquisition system and triaxial ICP accelerometers. Key measurement points were arranged on the bearing housings of the secondary transmission stage and the planetary gearbox casing (indicated by red circles in Fig. 26) to capture vibration signals from the housing surfaces. Furthermore, a dial indicator was employed to precisely measure the coaxiality of the transmission shafts and the installation eccentricity of the gears. This step provided direct experimental input for quantifying the initial state errors of the system, particularly the eccentricity ratio (*e*_1_/*e*_2_) parameter used in the simulation analysis, thereby ensuring the comparability of conditions between the simulation and experimental results.

### Comparison and analysis of the results

**Fig. 27:**
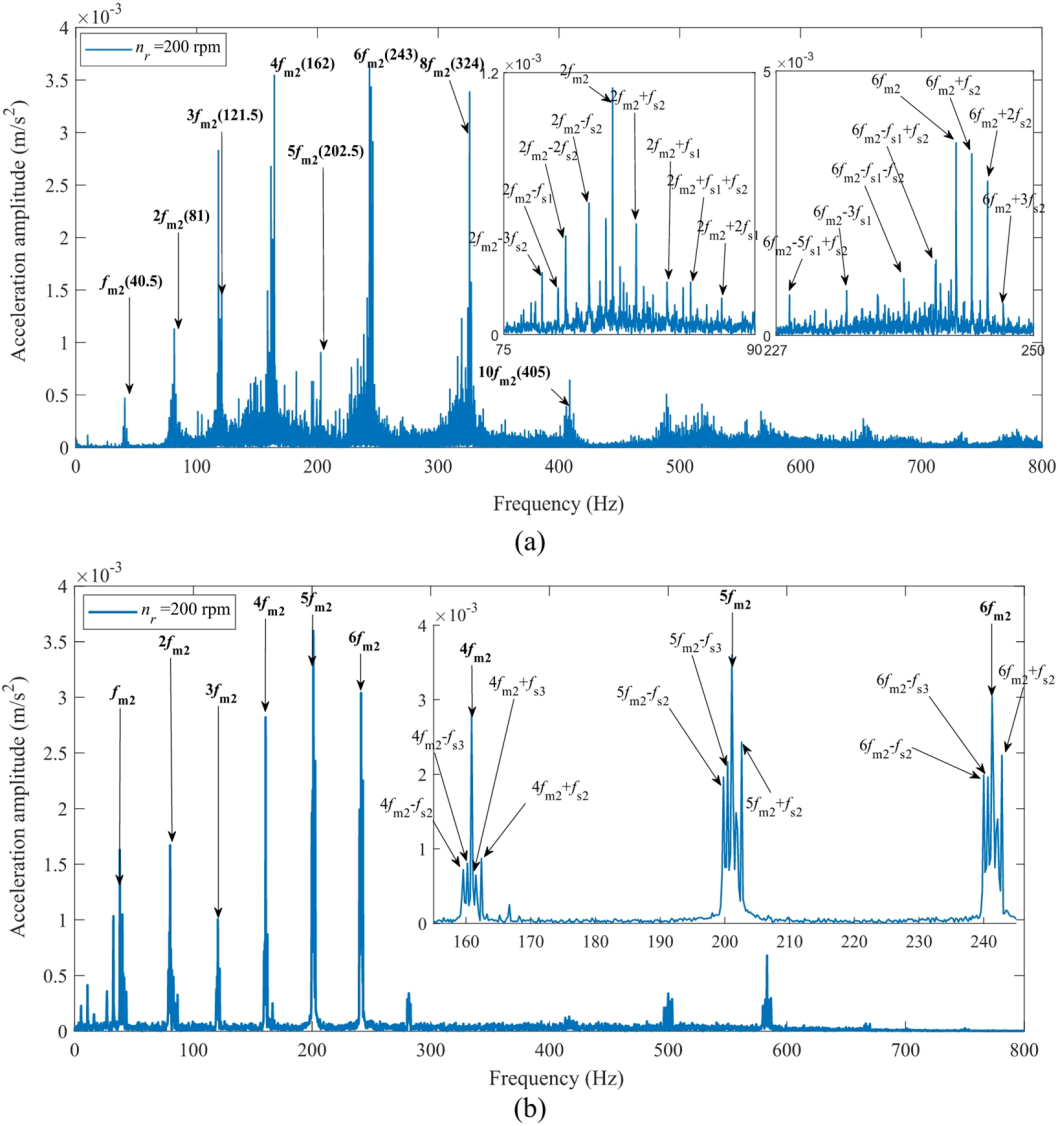
Spectrum of the second-stage transmission in the fixed-axis gearbox at an input speed of 200 rpm: (**a**) Experimental values; (**b**) Simulation results.

Figure 27 presents a comparison of the acceleration spectra from the second-stage transmission system in the fixed-axis gearbox at an input speed of 200 rpm. Figure 27(a) shows the experimental values, while Fig. 27(b) displays the simulation results. The simulation data were derived from the simulated signal of gear *g*_3_ mounted on the second-stage transmission shaft, as its location is closer to the test point on the second-stage bearing housing. In Fig. 27, *f*_*m*2_ denotes the meshing frequency of the second-stage gear transmission, and *f*_*si*_ represents the rotational frequencies of the transmission shafts at each stage (*i* = 1, 2, 3).

The comparative analysis of Fig. 27 demonstrates a high degree of consistency in the core spectral features between the simulated and measured acceleration spectra of the second-stage transmission system at an input speed of 200 rpm. Both methods accurately captured the spectral structure dominated by the second-stage meshing frequency (*f*_*m*2_≈40.5 Hz) and its significant harmonics (e.g., 3*f*_*m*2_ ≈ 121.5 Hz, 4*f*_*m*2_ ≈ 162 Hz, 6*f*_*m*2_ ≈ 243 Hz). The corresponding peak amplitudes are both on the order of 4 × 10^− 3^ m/s², thereby validating the effectiveness of the simulation model.

The experimental spectra exhibit more sideband components (e.g., 2*f*_*m*2_ ± 2*f*_*s*2_, 2*f*_*m*2_ − 3*f*_*s*2_, etc.) compared to the simulation results. This is primarily because the simulation employs a first-order Taylor series expansion to approximate the modulation effects induced by gear eccentricity, thereby neglecting higher-order sideband terms. However, the amplitudes of these higher-order sidebands observed in the experiment are significantly lower than those of the first-order sidebands and the main meshing frequency peaks, confirming the validity of this simplification. This approach ensures the prediction accuracy of key dynamic characteristics while significantly improving computational efficiency, thereby providing a more efficient computational tool for engineering design and parameter optimization.

**Fig. 28:**
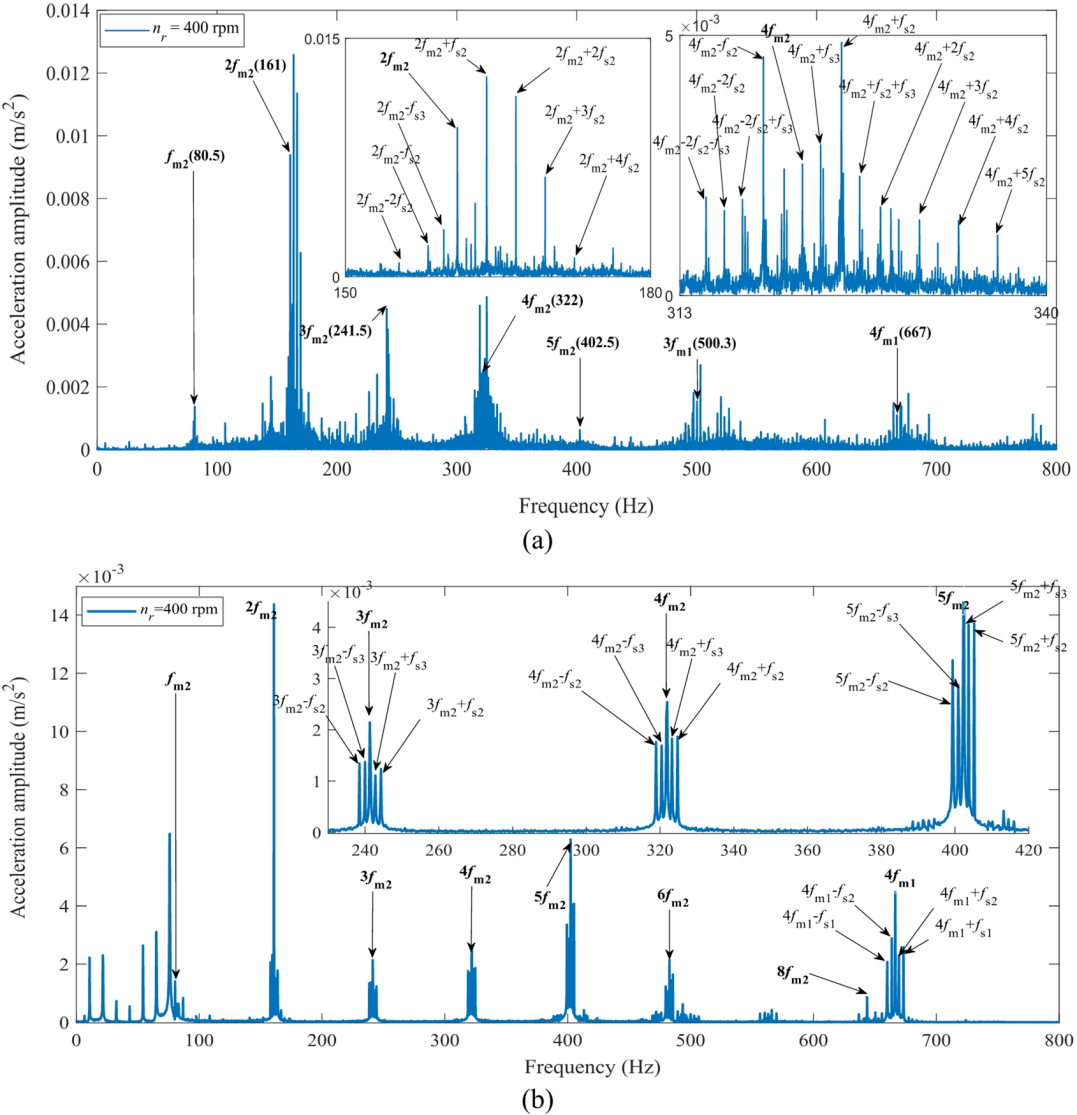
Vibration spectrum of the second-stage transmission in the fixed-axis gearbox at a motor speed of 400 rpm: (**a**) Experimental results, (**b**) Simulation results.

**Fig. 29:**
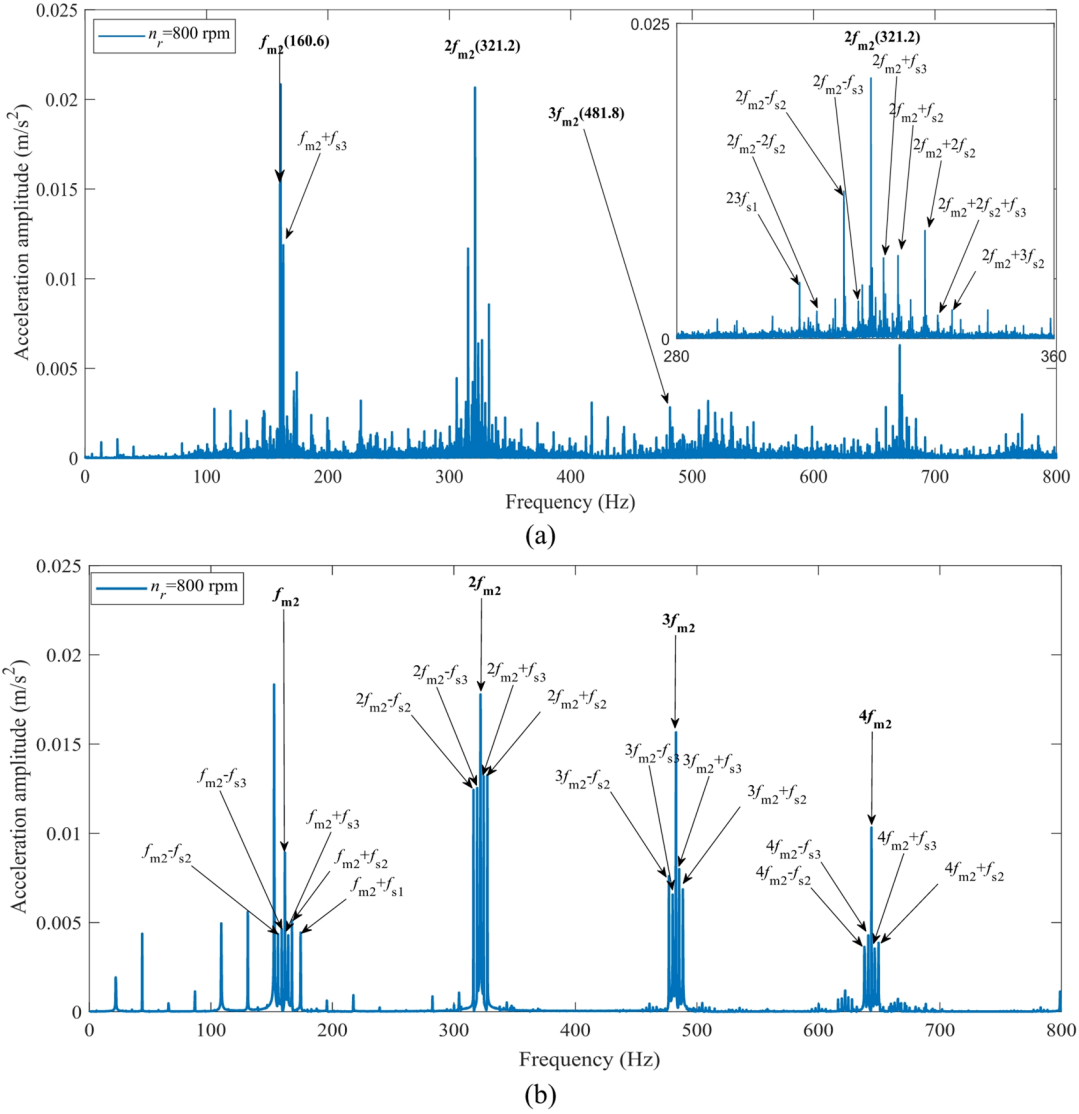
Vibration spectrum of the second-stage transmission in the fixed-axis gearbox at a motor speed of 800 rpm: (**a**) Experimental results, (**b**) Simulation results.

Figures 28 and 29 show the comparative results of the acceleration spectra for the second-stage transmission system in the fixed-axis gearbox at input speeds of 400 rpm and 800 rpm, respectively. A comparison of the results demonstrates that the proposed simulation model can accurately reproduce the core spectral characteristics measured in experiments under different rotational speeds in Figs. 28 and 29. Under both 400 rpm and 800 rpm conditions, the simulation accurately predicted the spectral structure dominated by the second-stage meshing frequency (*f*_*m*2_) and its harmonics (2*f*_*m*2_, 3*f*_*m*2_, 4*f*_*m*2_, etc.). The errors in the dominant peak frequencies were all less than 0.5 Hz, validating the model’s effectiveness in characterizing the fundamental excitation characteristics of the system.

Similarly, explainable differences exist between the simulation and experimental results regarding the sideband distribution. A greater number of higher-order sidebands (e.g., *if*_*m*2_±2*f*_*s*2_, *if*_*m*2_+3*f*_*s*2_, *if*_*m*2_+4*f*_*s*2_, where *i* = 1, 2, 3) appear in the experimental spectrum. However, their amplitudes are significantly lower than those of the dominant peaks and the first-order sidebands. This observation confirms the rationality of adopting a first-order approximation in the simulation, where higher-order terms are neglected. This simplification maintains computational efficiency without compromising the primary dynamic conclusions.

## Conclusions

This study employs a systematic approach integrating theoretical modeling, numerical simulation, and experimental validation to thoroughly investigate the dynamic coupling mechanisms of geometric eccentricity in the MGTS. It specifically elucidates the influence of eccentricity errors on system vibrational characteristics through the combined effects of stiffness modulation and dynamic excitations. The main conclusions are as follows:

(1) An analytical model for three-path stiffness modulation was established, accounting for center distance fluctuation, pressure angle reconstruction, and transient contact ratio. This model quantified the contribution weight of each physical path. The results demonstrate that geometric eccentricity significantly amplifies the fluctuation amplitude of the time-varying mesh stiffness by altering the line-of-action position and load distribution. The combination of a highly asymmetric eccentricity configuration (*e*_1_/*e*_2_ = 0.1 or 10) and an anti-phase operating condition (Δ*ϕ* = π) induces the most potent stiffness modulation effect, with the stiffness fluctuation amplitude reaching up to 3.5 × 10⁷ N/m. In contrast, a symmetric eccentricity structure (*e*_1_/*e*_2_ ≈ 1) coupled with an optimized phase difference (Δ*ϕ* ≈ π/2 or 3π/2) can effectively suppress stiffness fluctuations, reducing the amplitude to below 0.5 × 10⁷ N/m.

(2) A strong nonlinear coupling effect between the eccentricity ratio (*e*_1_/*e*_2_) and the initial phase difference (Δ*ϕ*) on the system’s dynamic response was discovered. The amplitude of center distance fluctuation (Δ*a*), the peak-to-peak value of modulated stiffness (Δ*k*_*e*_), and the eccentricity excitation (*F*_ecc_) all reached their maximum values at Δ*ϕ* = π and their minimum values at *e*_1_/*e*_2_ = 1. Multi-parameter collaborative optimization provides clear design guidelines for vibration control in transmission systems: the high-risk parameter combination of “asymmetry + anti-phase” (*e*_1_/*e*_2_ < < 1 and Δ*ϕ* = π) must be avoided. To achieve high performance, a symmetric eccentricity design (*e*_1_/*e*_2_ ≈ 1) should be prioritized, with the phase difference set around Δ*ϕ* ≈ π/2 or 3π/2, thereby suppressing vibration excitation at the source.

(3) Vibration energy exhibits significant attenuation characteristics along the transmission chain, with the vibration response of the fixed-axis stage (high-speed stage) gears being markedly higher than that of the planetary stage. The peak acceleration of gear *g*_2_ in the fixed-axis stage (≈ 28 m/s²) is approximately four times greater than that of the sun gear (≈ 7 m/s²), indicating that the high-speed stage is the primary vibration source and should be the foremost target for vibration control. Furthermore, eccentric errors disrupt the load sharing characteristics of the PGS, leading to significant differences in the load fluctuation factors among the planets (e.g., the fluctuation factor for *F*_*sp*1_ ranges from 1.12 to 1.25, while for *F*_*sp*3_ it can reach 0.9 to 1.8).

(4) A three-stage transmission test rig was constructed, and experimental tests alongside model validation were conducted under different rotational speeds (200 ~ 800 rpm). The experimental results show high consistency with the simulation results in the main spectral characteristics (meshing frequencies and their harmonics), with frequency errors of less than 0.5 Hz, thereby validating the effectiveness of the developed dynamic model. The additional higher-order sidebands observed in the experimental spectra primarily originate from the first-order Taylor series approximation adopted in the simulation for modeling the gear eccentricity modulation effects, which neglects higher-order modulation terms. However, the amplitudes of these higher-order sidebands in the experiments are considerably lower than those of the first-order sidebands and the dominant peaks, confirming the rationality of the simplified approach used in this study.

(5) This study ultimately constructs a vibration-performance-based “safe design region” for parameter selection. The area defined by an eccentricity ratio of *e*_1_/*e*_2_ ≈ 0.5 ~ 2 and a phase difference of Δ*ϕ* ≈ 0 ~ 1.5 rad represents the optimal design domain, enabling the achievement of globally minimal vibration. The green region covers a broader parameter range and exhibits strong robustness, providing a design window that balances manufacturing economy with performance stability. For the design of precision transmission systems, it is recommended to prioritize a symmetric eccentricity structure (*e*_1_/*e*_2_ ≈ 1) and achieve vibration suppression by optimizing the phase difference (keeping Δ*ϕ* away from π).

## Data Availability

The datasets generated and analyzed during this study, including dynamic simulation results and experimental measurements, are available from the corresponding author upon reasonable request. The specific parameters employed in the dynamic model are comprehensively listed in the article’s tables.

## References

[1] Badihi, H., Zhang, Y., Jiang, B., Pillay, P. & Rakheja, S. A comprehensive review on signal-based and model-based condition monitoring of wind turbines: fault diagnosis and lifetime prognosis. *Proc. IEEE*. **110** (6), 754–806 (2022).

[2] Wang, X. & Xia, P. Novel modeling and vibration analysis method on a helicopter drive train system. *AIAA J.* **60** (7), 4288–4301 (2022).

[3] Zhang, R., Zhou, J. & Wei, Z. Study on transmission error and torsional stiffness of RV reducer under wear. *J. Mech. Sci. Technol.* **36** (8), 4067–4081 (2022).

[4] Gu, X. & Velex, P. A dynamic model to study the influence of planet position errors in planetary gears. *J. Sound Vib.* **331** (20), 4554–4574 (2012).

[5] Jordan, J. M., De Smet, B., Blockmans, B. & Desmet, W. A misaligned formulation for planetary gears with analytical 3D contact characterization. *Nonlinear Dyn.* **112** (19), 16811–16836 (2024).

[6] Zhao, B., Huangfu, Y., Ma, H., Zhao, Z. & Wang, K. The influence of the geometric eccentricity on the dynamic behaviors of helical gear systems. *Eng. Fail. Anal.* **118**, 104907 (2020).

[7] Liu, W., Zhao, H., Lin, T., Gao, B. & Yang, Y. Vibration characteristic analysis of gearbox based on dynamic excitation with eccentricity error. *J. Mech. Sci. Technol.* **34** (11), 4545–4562 (2020).

[8] Duan, L., Wang, L., Du, W., Shao, Y. & Chen, Z. Analytical method for time-varying meshing stiffness and dynamic responses of modified spur gears considering pitch deviation and geometric eccentricity. *Mech. Syst. Signal Process.* **218**, 111590 (2024).

[9] Elforjani, M., Mba, D. & Salihu, B. Experimental monitoring of eccentric gears with different mechanical conditions. *Appl. Acoust.* **197**, 108903 (2022).

[10] Ohta, H., Yamakawa, A. & Katayama, Y. Effects of eccentricity on transmission errors of trochoidal gears. *ASME J. Tribology*. **134** (1), 011102 (2012).

[11] Li, J., Chen, H., Wang, X. B. & Yang, Z. X. A comprehensive gear eccentricity dataset with multiple fault severity levels: Description, characteristics analysis, and fault diagnosis applications. *Mech. Syst. Signal Process.* **224**, 112068 (2025).

[12] Chaari, F., Fakhfakh, T., Hbaieb, R., Louati, J. & Haddar, M. Influence of manufacturing errors on the dynamic behavior of planetary gears. *Int. J. Adv. Manuf. Technol.* **27** (7), 738–746 (2006).

[13] Inalpolat, M. & Kahraman, A. A dynamic model to predict modulation sidebands of a planetary gear set having manufacturing errors. *J. Sound Vib.* **329** (4), 371–393 (2010).

[14] Bodas, A. & Kahraman, A. Influence of carrier and gear manufacturing errors on the static load sharing behavior of planetary gear sets. *JSME Int. J. C*. **47** (3), 908–915 (2004).

[15] Gu, X. & Velex, P. On the dynamic simulation of eccentricity errors in planetary gears. *Mech. Mach. Theory*. **61**, 14–29 (2013).

[16] Leque, N. & Kahraman, A. A three-dimensional load sharing model of planetary gear sets having manufacturing errors. *J. Mech. Des.* **139** (3), 033302 (2017).

[17] Hu, Y., Ryali, L., Talbot, D. & Kahraman, A. A theoretical study of the overall transmission error in planetary gear sets. *Proc. Institution Mech. Eng. Part. C: J. Mech. Eng. Sci.* **233** (21-22), 7200–7211 (2019).

[18] Ligata, H., Kahraman, A. & Singh, A. An experimental study of the influence of manufacturing errors on the planetary gear stresses and planet load sharing. *J. Mech. Des.* **130** (4), 041701 (2008).

[19] Park, J. et al. Model-based fault diagnosis of a planetary gear: A novel approach using transmission error. *IEEE Trans. Reliab.* **65** (4), 1830–1841 (2016).

[20] Peng, D., Smith, W. A., Borghesani, P., Randall, R. B. & Peng, Z. Comprehensive planet gear diagnostics: use of transmission error and mesh phasing to distinguish localised fault types and identify faulty gears. *Mech. Syst. Signal Process.* **127**, 531–550 (2019).

[21] Mo, S., Zhang, Y., Wu, Q., Matsumura, S. & Houjoh, H. Load sharing behavior analysis method of wind turbine gearbox in consideration of multiple-errors. *Renew. Energy*. **97**, 481–491 (2016).

[22] Sun, W. et al. A study on load-sharing structure of multi-stage planetary transmission system. *J. Mech. Sci. Technol.* **29** (4), 1501–1511 (2015).

[23] Zhai, H., Zhu, C., Song, C., Liu, H. & Bai, H. Influences of carrier assembly errors on the dynamic characteristics for wind turbine gearbox. *Mech. Mach. Theory*. **103**, 138–147 (2016).

[24] Nejad, A. R. et al. Effects of floating sun gear in a wind turbine’s planetary gearbox with geometrical imperfections. *Wind Energy*. **18** (12), 2105–2120 (2015).

[25] Xie, F., Sun, Y., Wu, S., Zhang, Q. & Li, X. Research on load sharing performance of wind turbine gearbox involving multiple-errors and tooth crack. *J. Mech. Sci. Technol.* **36** (9), 4395–4408 (2022).

[26] Lu, W. & Zhang, Y. Research on the effect of tooth root defects on the motor-multistage gear transmission system. *Mech. Based Des. Struct. Mach.* **52** (3), 1561–1583 (2024).

[27] Zhang, X., Tang, X. & Yang, W. Analysis of transmission error and load distribution of a Hoist two-stage planetary gear system. *Proc. Institution Mech. Eng. Part. K: J. Multi-body Dynamics*. **233** (1), 3–16 (2019).

[28] Zhang, Y., Wang, Q., Ma, H., Huang, J. & Zhao, C. Dynamic analysis of three-dimensional helical geared rotor system with geometric eccentricity. *J. Mech. Sci. Technol.* **27** (11), 3231–3242 (2013).

[29] Lin, J. & Parker, R. G. Analytical characterization of the unique properties of planetary gear free vibration. *J. Vib. Acoust.* **121** (3), 316–321 (1999).

[30] Wei, S., Han, Q., Peng, Z. & Chu, F. Dynamic analysis of parametrically excited system under uncertainties and multi-frequency excitations. *Mech. Syst. Signal Process.* **72–73**, 762–784 (2016).

[31] Pohrt, R. & Popov, V. L. Contact mechanics of rough spheres: crossover from fractal to Hertzian behavior. *Adv. Tribology*. **2013** (1), 974178 (2013).

